# A rhinopithecus swarm optimization algorithm for complex optimization problem

**DOI:** 10.1038/s41598-024-66450-x

**Published:** 2024-07-07

**Authors:** Guoyuan Zhou, Dong Wang, Guoao Zhou, Jiaxuan Du, Jia Guo

**Affiliations:** 1 College of Informatics, Huazhong Agricultural University, Wuhan, 430070 China; 2 School of Automation, Wuhan University of Technology, Wuhan, 430070 China; 3 Hubei Key Laboratory of Digital Finance Innovation, Hubei University of Economics, Wuhan, 430205 China; 4 School of Information Engineering, Hubei University of Economics, Wuhan, 430205 China; 5 Hubei Internet Finance Information Engineering Technology Research Center, Wuhan, 430205 China

**Keywords:** Computational science, Computer science, Information technology

## Abstract

This paper introduces a novel meta-heuristic algorithm named Rhinopithecus Swarm Optimization (RSO) to address optimization problems, particularly those involving high dimensions. The proposed algorithm is inspired by the social behaviors of different groups within the rhinopithecus swarm. RSO categorizes the swarm into mature, adolescent, and infancy individuals. Due to this division of labor, each category of individuals employs unique search methods, including vertical migration, concerted search, and mimicry. To evaluate the effectiveness of RSO, we conducted experiments using the CEC2017 test set and three constrained engineering problems. Each function in the test set was independently executed 36 times. Additionally, we used the Wilcoxon signed-rank test and the Friedman test to analyze the performance of RSO compared to eight well-known optimization algorithms: Dung Beetle Optimizer (DBO), Beluga Whale Optimization (BWO), Salp Swarm Algorithm (SSA), African Vultures Optimization Algorithm (AVOA), Whale Optimization Algorithm (WOA), Atomic Retrospective Learning Bare Bone Particle Swarm Optimization (ARBBPSO), Artificial Gorilla Troops Optimizer (GTO), and Harris Hawks Optimization (HHO). The results indicate that RSO exhibited outstanding performance on the CEC2017 test set for both 30 and 100 dimension. Moreover, RSO ranked first in both dimensions, surpassing the mean rank of the second-ranked algorithms by 7.69% and 42.85%, respectively. Across the three classical engineering design problems, RSO consistently achieves the best results. Overall, it can be concluded that RSO is particularly effective for solving high-dimensional optimization problems.

## Introduction

In recent years, more and more optimization problems, such as route planning^1–3^, resource allocation^4–6^, and other real-world problems, have received increased attention from scholars. These optimization problems have an optimal solution that comprises a combination of multiple variables. Researchers aim to find the optimal solution as much as possible by using optimization algorithms.

Optimization algorithms play a crucial role in practical applications. In manufacturing^7–10^, optimization algorithms can be employed to optimize production processes, reduce costs, and improve efficiency. In the field of transportation planning^11,12^, they can help optimize traffic flow, reduce congestion, and improve road usage. In healthcare^13,14^, these algorithms can be utilized to optimize the allocation of hospital resources and improve the efficiency of healthcare services. Another important application area is financial and investment management^15,16^. Optimization algorithms can help investors find the best asset allocation in their portfolios to minimize risk and achieve expected returns. Furthermore, optimization algorithms have been applied in the development of Deep Learning (DL) and Machine Learning (ML)^17–19^. In ML and DL, optimization algorithms can improve the performance of models by tuning hyperparameters.

There are various types of optimization algorithms. These include gradient descent algorithms^20^ based on mathematics, dynamic programming algorithms^21^ based on operations research, and optimization algorithms^22–24^ based on heuristics. Among these, meta-heuristic optimization algorithms have a unique advantage in solving more complex optimization problems. They do not rely on specific problem knowledge but instead explore and exploit the solution space to find the optimal or near-optimal solution. These algorithms are usually designed based on natural phenomena and group behavior. Dehghani^25^ introduced the Coati Optimization Algorithm (COA), which mimics coati behavior in nature. Al-Betar^26^ proposed a novel nature-inspired swarm-based optimization algorithm called the Elk Herd Optimizer (EHO), inspired by the breeding process of elk herds. Zhao^27^ proposed the Sea-Horse Optimizer (SHO), inspired by the behavior of seahorses, mainly mimicking their movement, predation, and breeding behaviors. Hashim^28^ mathematically modeled the foraging and reproduction behaviors of snakes to present the Snake Optimizer (SO). Abdollahzadeh^29^ introduced the African Vultures Optimization Algorithm (AVOA), inspired by the foraging and navigation behaviors of African vultures. Zhong^30^ designed the Beluga Whale Optimization (BWO) algorithm, inspired by the behaviors of beluga whales. MiarNaeimi^31^ proposed the Horse Herd Optimization Algorithm (HOA), which imitates the social behaviors of horses at different ages. Heidari^32^ introduced the Harris Hawks Optimization (HHO) algorithm, inspired by the cooperative behavior and chasing style of Harris hawks in nature.

To further improve the performance of existing optimization algorithms, researchers have introduced several enhancements. Based on Bare-Bone Particle Swarm Optimization (BBPSO), Zhou^33^ proposed an atomic retrospective learning BBPSO (ARBBPSO) algorithm by introducing the renewal strategy of motion around nuclei and the retrospective learning strategy. Additionally, mutation methods^34,35^ are employed to enhance the exploration capabilities of optimization algorithms. Abed-alguni^36^ introduced the island-based Cuckoo Search with Polynomial Mutation (iCSPM), which replaces the Lévy flight method in the original Cuckoo Search with the highly disruptive polynomial mutation method.

Although these meta-heuristic optimization algorithms can solve some optimization problems well, high-dimensional optimization problems remain a challenge. High-dimensional optimization problems usually involve a large number of parameters or variables, leading to a dramatic increase in the dimension of the solution space. In high-dimensional space, the increase in the width and depth of the solution space results in a higher number of locally optimal solutions, making it more difficult to escape from the local optimum. Additionally, high-dimensional optimization problems lead to the “curse of dimensionality”^37^. As the dimension increases, the distance between valid data points becomes extremely dispersed, thus affecting the convergence speed and stability of the algorithm.

When solving high-dimensional optimization problems, although the Honey Badger Algorithm (HBA)^38^ and the Salp Swarm Algorithm (SSA)^39^ converge quickly in local search, they tend to fall into local optima due to insufficient global search capability. Due to their weak exploitation abilities, the Chimp Optimization Algorithm (ChOA)^40^ and the Whale Optimization Algorithm (WOA)^41^ show low convergence accuracy in high-dimensional optimization problems. The Artificial Gorilla Troops Optimizer (GTO)^42^ and the Dung Beetle Optimizer (DBO)^43^ struggle to escape local optima due to a lack of local search capability when dealing with high-dimensional problems.

To address these challenges, we propose a novel swarm-based Rhinopithecus Swarm Optimization (RSO), inspired by the social behaviors of rhinopithecus. In the RSO, we introduce three distinct search strategies: vertical migration, concerted search, and mimicry. Additionally, RSO divides the swarm into different categories, with each category allocated a corresponding search strategy and specific learning objectives. This approach enables individuals in the swarm to explore the solution space from multiple perspectives, thus increasing the coverage of the search space. Consequently, the proposed algorithm can effectively overcome high-dimensional optimization challenges.

The main contributions of this paper are as follows:

1. We propose a novel Rhinopithecus Swarm Optimization (RSO) algorithm, which can solve optimization problems and complex engineering design problems well.
2. Three different search strategies of RSO are devised based on the social behaviors of different groups in the swarm. These strategies aim to escape local optima by enhancing global and local search capabilities.
3. RSO is evaluated against eight well-known optimization algorithms. The results verify its superior performance in solving optimization challenges and complex engineering design problems, especially in high-dimensional optimization problems.

The rest of this paper is organized as follows: section "Methods" introduces the details of RSO. Section "Experiments" shows the results and analysis of the simulation experiments. Section "Engineering design problems" introduces the engineering problems and the experimental results. Section "Conclusions and future works" summarizes this work and details some possible future directions.

## Methods

Optimization problems become more challenging with increasing dimensionality. In high-dimensional optimization problems, the number of local optimal solutions increases with dimension, making it easier for traditional optimization algorithms to fall into local optima. Therefore, the challenge is to improve the efficiency of searching the large-scale solution space to overcome the curse of dimensionality. To better address these problems, we have developed a novel Rhinopithecus Swarm Optimization (RSO) algorithm inspired by the social behavior of rhinopithecus swarm, including vertical migration, concerted search, and mimicry. In this section, we discuss some details of rhinopithecus behavior and the RSO.

### Behavior of rhinopithecus

Rhinopithecus is an agile and intelligent animal, excelling in leaping and climbing among the canopy and often foraging while traversing trees. Rhinopithecus exhibit strong adaptability, with their habitat spanning various regions from low to high altitudes. Due to the influence of external factors such as ambient temperature and food distribution, the rhinopithecus swarm frequently displays vertical migration tendencies. Within the swarm, individuals of various age structures play distinct roles during these migration events. Mature rhinopithecus are responsible for guiding the migration direction of the swarm. Adolescent rhinopithecus, being less robust than the mature individuals, typically learn survival skills by emulating mature rhinopithecus and generally seek habitats around them. Infant rhinopithecus lack the ability to search for survival resources and usually rely on older individuals to acquire these resources for them.

### Rhinopithecus swarm optimization algorithm

Inspired by the social behavior of the rhinopithecus swarm, we have introduced the Rhinopithecus Swarm Optimization (RSO) algorithm. The ways the rhinopithecus swarm searches for survival positions provide new strategies for the optimization algorithm, enhancing its exploration and exploitation capabilities. In RSO, the search space represents the natural habitat of rhinopithecus. The survival position of an individual in the search space corresponds to a solution of the optimization algorithm. Individuals occupy superior or inferior survival positions based on their survival experience, with older individuals typically occupying better positions.

**Figure 1:**
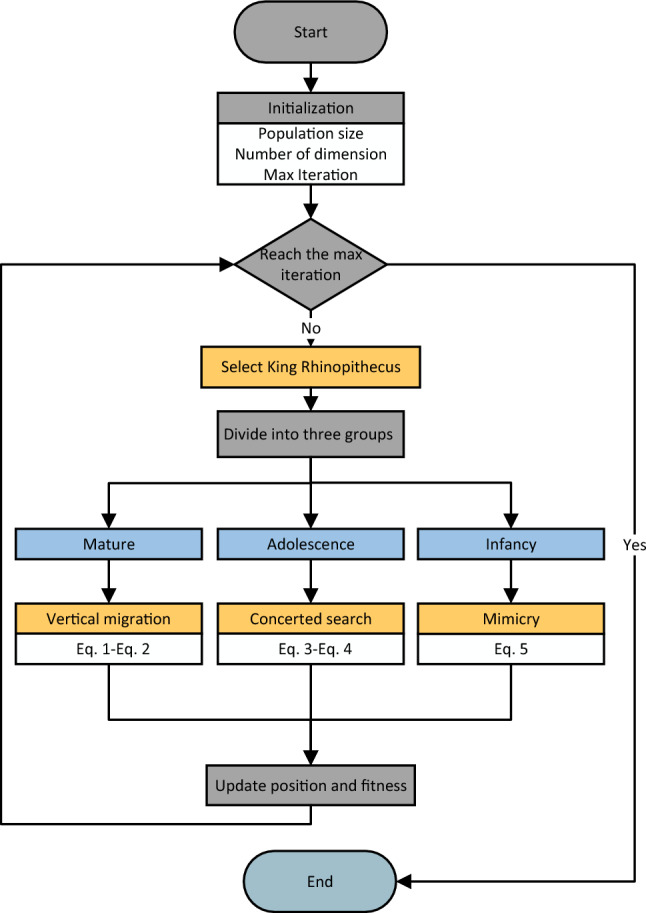
The flowchart of RSO.

In RSO, we categorize the top 40% of individuals as mature rhinopithecus, those ranking between 40 and 70% as adolescent rhinopithecus, and the remaining individuals as infancy rhinopithecus based on their survival positions. The individual with the best survival position in the rhinopithecus swarm is referred to as the king rhinopithecus, who usually emerges from the mature individuals. The king rhinopithecus and the mature individuals jointly lead the migration of the groups. The flowchart and pseudo-code of RSO is shown in Fig. 1 and Algorithm 1.

**Algorithm 1:**
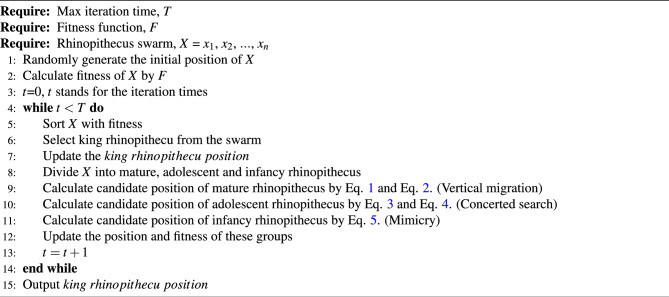
Pseudo-code of RSO

### Vertical migration

The vertical migratory behavior of rhinopithecus plays a crucial role in the survival of the swarm. Typically, the rhinopithecus swarm periodically migrates between lower and higher altitudes depending on food distribution and climatic conditions. At low temperatures, rhinopithecus prefer to be active at lower altitudes, where the lower altitude reduces the physiological stress of cold temperatures and food is relatively more abundant. At other temperatures, the rhinopithecus swarm displays the opposite migratory trend, preferring to utilize higher altitude areas where food is relatively higher quality.

Researchers have found that rhinopithecus possess a certain degree of spatial cognition. They may be able to memorize positions they have inhabited and incorporate past experience and foraging strategies to guide migration. Mature and king rhinopithecus usually have richer experience and spatial cognition of the migration process, playing a leadership role in the swarm migration. These individuals retain optimal positions for two temperature conditions, as shown in Eq. (1). During the next migration, they search for a new survival position by integrating previous survival experience. This approach helps the rhinopithecus swarm select better positions effectively.

1 $$\begin{aligned} KingR&= \begin{bmatrix} KingR, KingR_{1}, KingR_{2} \end{bmatrix} \\ MR&= \begin{bmatrix} MR, MR_{1}, MR_{2} \end{bmatrix} \end{aligned}$$

where *KingR* stands for the king rhinopithecus, *MR* stands for the mature rhinopithecus.

Each time the mature rhinopithecus search for a candidate position, they try to move towards the position of the king rhinopithecus. Their candidate solution is calculated by Eq. (2).

2 $$\begin{aligned} \begin{aligned} &\alpha = \frac{{KingR_{a} + MR_{b}}}{2} \\&\beta = |KingR_{a} - MR_{b}|\\&CandiMR = Gausi(\alpha ,\beta ) \end{aligned}&\quad \begin{aligned} a, b \in [0, 2] \end{aligned} \end{aligned}$$

where $KingR_{a}$ stand for the locations of the king rhinopithecus, the $MR_{b}$ stand for the locations of the mature rhinopithecus. $Gausi(\alpha ,\beta )$ was a function value that generates random from a Gaussian distribution with an expectation of $\alpha$ and a variance of $\beta$.

### Concerted search

In the rhinopithecus swarm, adolescent rhinopithecus are in the growth stage. Although they have some ability to search for migratory positions, they lack the experience of mature and king rhinopithecus. Consequently, adolescent rhinopithecus often exhibit uncertainty in choosing search paths and migration positions. In such cases, they actively seek guidance from mature rhinopithecus and the king rhinopithecus, relying on their environmental cognition and survival experience to make decisions. Typically, adolescent rhinopithecus communicate information about their historical positions to the king and mature rhinopithecus. The king and mature individuals then use their own experience to guide the adolescents in making judgments. In RSO, adolescent rhinopithecus are set to communicate two historical positions to the king and mature rhinopithecus, respectively.

3 $$AR = \begin{bmatrix} AR_{1}, AR_{2} \end{bmatrix}$$

where *AR* stands for the adolescent rhinopithecus.

The king Rhinopithecus and the mature individuals provide the adolescent individuals with their respective advice. The adolescent rhinopithecus consider both suggestions equally and synthesize the opinions to generate a candidate solution, which is calculated using Eq. (4). By leveraging the survival experience of the older individuals, adolescent rhinopithecus can select better positions. This strategy allows RSO to explore the solution space more effectively, thereby improving its ability to escape from local optima.

4 $$\begin{aligned} \begin{aligned} \gamma &= \frac{{KingR_{a} + AR_{c}}}{2} \\ \epsilon &= \frac{{MR_{b} + AR_{c}}}{2} \\ \delta&= |KingR_{a} - AR_{c} |\\ \zeta&= |MR_{b} - AR_{c} |\\ CandiAR&= \frac{{Gausi(\gamma ,\delta ) + Gausi(\epsilon ,\zeta )}}{2} \end{aligned}&\begin{aligned} \quad a, b \in [0, 2] \\ \quad c \in [0, 1] \end{aligned} \end{aligned}$$

where $KingR_{a}$ stand for the locations of the king rhinopithecus, the $MR_{b}$ stand for the locations of the mature rhinopithecus, the $AR_{c}$ stand for the locations of the adolescent rhinopithecus. $Gausi(\gamma ,\delta )$ was a function value that generates random from a Gaussian distribution with an expectation of $\gamma$ and a variance of $\delta$. $Gausi(\epsilon ,\zeta )$ was a function value that generates random from a Gaussian distribution with an expectation of $\epsilon$ and a variance of $\zeta$.

### Mimicry

Infant rhinopithecus are usually in the early stages of learning and development. They have not fully mastered the elements of their environment and survival skills, making it very difficult for them to find suitable positions for migration. During this vital developmental stage, infancy individuals depend on other group members, especially adolescent and mature rhinopithecus, to lead them in their migration. In the searching process, infancy rhinopithecus communicate information about their position to these older individuals in various ways. Adolescent and mature rhinopithecus understand the habits of the infancy individuals based on this positional information. Thus, they can better integrate their own experience to guide the infancy rhinopithecus during migration.

In RSO, after receiving guidance from mature and adolescent rhinopithecus, the infancy individuals consider the suggestions from both groups and determine their candidate positions through comprehensive consideration, calculated using Eq. (5). This group-assisted approach helps infancy rhinopithecus navigate the growth stage seamlessly, allowing them to learn survival skills by imitating the search strategies of older individuals. This strategy enhances the ability to exchange information between different groups, which in turn improves the local search capability of RSO.

5 $$\begin{aligned} \begin{aligned} \eta &= \frac{{MR_{b} + IR}}{2} \\ \iota&= \frac{{AR_{c} + IR}}{2} \\ \theta&= |MR_{b} - IR|\\ \kappa&= |AR_{c} - IR |\\ CandiAR&= \frac{{Gausi(\eta ,\theta ) + Gausi(\iota ,\kappa )}}{2} \end{aligned}&\begin{aligned} \quad b \in [0, 2] \\ \quad c \in [0, 1] \end{aligned} \end{aligned}$$

where $MR_{b}$ stand for the locations of the mature rhinopithecus, the $AR_{c}$ stand for the locations of the adolescent rhinopithecus, *IR* stands for the position of the infancy rhinopithecus. $Gausi(\eta ,\theta )$ was a function value that generates random from a Gaussian distribution with an expectation of $\eta$ and a variance of $\theta$. $Gausi(\iota ,\kappa )$ was a function value that generates random from a Gaussian distribution with an expectation of $\iota$ and a variance of $\kappa$.

### Consent to participate

All authors participated in the manuscript and agreed to participate in it.æ

## Experiments

The CEC2017 function set is widely used to measure the comprehensive performance of optimization algorithms. All functions in the test set have undergone rotation and displacement, increasing the difficulty for optimization algorithms to find the optimum. The test set contains 29 test functions, each available in four dimensions: 10, 30, 50, and 100. The difficulty of solving these problems increases with the dimension. Based on the specific properties of the functions in the search space, they can be categorized into Unimodal Functions $(F_1-F_2)$, Simple Multimodal Functions ($F_3-F_9$), Hybrid Functions ($F_{10}-F_{19}$), and Composite Functions ($F_{20}-F_{29}$).

Table 1 shows the details of the CEC2017 benchmark functions. Unimodal Functions have only one significant optimum in the search space, making the optimization target clear. Simple Multimodal Functions contain multiple local optima, increasing the probability of falling into a local optimum. Hybrid Functions combine different properties such as linear and nonlinear components and low and high dimensional combinations, making the search space more complex. Composite Functions comprise combinations of simple functions, including linear combinations, nonlinear combinations, combinatorial functions, and transformation functions.

**Table 1:** Details of the CEC2017 benchmark functions.

Function number	Function name	Theoretical optimal
$F_{1}$	Shifted and Rotated Bent Cigar Function	100
$F_{2}$	Shifted and Rotated Zakharov Function	200
$F_{3}$	Shifted and Rotated Rosenbrock’s Function	300
$F_{4}$	Shifted and Rotated Rastrigin’s Function	400
$F_{5}$	Shifted and Rotated Expanded Scaffer’s F6 Function	500
$F_{6}$	Shifted and Rotated Lunacek Bi_Rastrigin Function	600
$F_{7}$	Shifted and Rotated Non-Continuous Rastrigin’s Function	700
$F_{8}$	Shifted and Rotated Levy Function	800
$F_{9}$	Shifted and Rotated Schwefel’s Function	900
$F_{10}$	Hybrid Function 1 ($\hbox {N}=3$)	1000
$F_{11}$	Hybrid Function 2 ($\hbox {N}=3$)	1100
$F_{12}$	Hybrid Function 3 ($\hbox {N}=3$)	1200
$F_{13}$	Hybrid Function 4 ($\hbox {N}=4$)	1300
$F_{14}$	Hybrid Function 5 ($\hbox {N}=4$)	1400
$F_{15}$	Hybrid Function 6 ($\hbox {N}=4$)	1500
$F_{16}$	Hybrid Function 6 ($\hbox {N}=5$)	1600
$F_{17}$	Hybrid Function 6 ($\hbox {N}=5$)	1700
$F_{18}$	Hybrid Function 6 ($\hbox {N}=5$)	1800
$F_{19}$	Hybrid Function 6 ($\hbox {N}=6$)	1900
$F_{20}$	Composition Function 1 ($\hbox {N}=3$)	2000
$F_{21}$	Composition Function 1 ($\hbox {N}=3$)	2100
$F_{22}$	Composition Function 1 ($\hbox {N}=4$)	2200
$F_{23}$	Composition Function 1 ($\hbox {N}=4$)	2300
$F_{24}$	Composition Function 1 ($\hbox {N}=5$)	2400
$F_{25}$	Composition Function 1 ($\hbox {N}=5$)	2500
$F_{26}$	Composition Function 1 ($\hbox {N}=6$)	2600
$F_{27}$	Composition Function 1 ($\hbox {N}=6$)	2700
$F_{28}$	Composition Function 1 ($\hbox {N}=3$)	2800
$F_{29}$	Composition Function 1 ($\hbox {N}=3$)	2900

To verify the comprehensive ability of RSO to solve optimization problems, we performed tests with 30 and 100 dimension using the CEC2017 benchmark. In the study, we used a series of well-known optimization algorithms as the control group, including DBO, BWO, SSA, AVOA, WOA, ARBBPSO, GTO, and HHO. To ensure the reliability of the results, the control group algorithms used the same parameter settings as in their original papers. The proposed algorithm, RSO, is a parameter-free meta-heuristic algorithm.

The time complexity of RSO is $O(m \cdot n)$, where $m$ represents the population size and $n$ represents the number of iterations. The proposed algorithm evaluates each individual only once per iteration without additional computations, resulting in a relatively low time complexity.

Mean Error (ME) was used to measure the performance of the algorithms. ME is defined as the mean error over multiple experiments, where the error is calculated as $|Real_{optimal} - Theoretical_{optimal}|$. To minimize the impact of chance on the experimental results and performance analysis, we conducted 36 independent experiments for each test function. The errors of these experiments were averaged to obtain the ME. The results of the experiments are shown in Tables 2, 3, 4, 5, 6, 7, 8, 9 and 10. The Mean, Best, Median, Worst, Standard Deviation (Std), and $p-value$ of the 36 runs were recorded.

When the dimension was set to 30, out of 29 functions, RSO achieved 10 first rankings, 8 second rankings, 6 third rankings, 2 fourth rankings, 2 sixth rankings, and 1 eighth ranking. RSO obtained the highest number of first rankings among all algorithms, 3 more than ARBBPSO, which had the second-highest number. In each type of function in CEC2017, RSO outperformed nearly all the algorithms in the control group, especially for Simple Multimodal Functions, where RSO obtained 4 first rankings out of 7 test functions. However, the test results of RSO on Unimodal Functions were inferior to those of SSA and AVOA.

As the dimension was adjusted to 100, RSO still maintained outstanding performance, with the number of first rankings increasing to 19. While SSA received first rankings in only 6 functions, several algorithms did not achieve any first rankings. In all four types of CEC2017 functions, RSO showed significant advantages over the control group. In each category of test functions, the number of first rankings obtained by RSO was not less than that of the control group. It was only on $F_3$ of Simple Multimodal Functions that RSO ranked relatively behind.

**Table 2:** Experimental results of the RSO, DBO, BWO, SSA, AVOA, WOA, ARBBPSO, GTO and HHO for $F_1$ - $F_{8}$ with 30 dimension.

Function	Type	RSO	DBO	BWO	SSA	AVOA	WOA	ARBBPSO	GTO	HHO
$F_1$	Mean	2.195E+04	1.217E+04	3.781E+10	1.758E+03	6.241E+03	4.126E+04	2.095E+04	4.997E+03	5.702E+06
	Std	2.514E+04	7.919E+03	1.661E+09	3.455E+03	7.520E+03	4.543E+04	3.577E+04	4.206E+03	9.027E+05
	Worst	5.895E+04	2.084E+04	4.011E+10	9.855E+03	1.977E+04	1.488E+05	1.458E+05	1.801E+04	7.775E+06
	Median	5.697E+03	5.490E+03	3.749E+10	5.335E+01	2.567E+03	1.682E+04	4.827E+03	3.072E+03	5.999E+06
	Best	2.957E+02	1.609E+02	3.537E+10	5.737E+00	5.685E+00	1.682E+04	1.891E+01	1.616E+03	3.660E+06
	p-value	~	1.928E-01	1.253E-13	8.912E-09	1.758E-03	1.719E-04	2.278E-01	2.670E-03	1.947E-13
	Rank	6	4 ($\approx$)	9 (+)	1 (−)	3 (–)	7 (+)	5 ($\approx$)	2 (–)	8 (+)
$F_2$	Mean	2.193E-04	2.134E+24	2.131E+41	1.481E-04	8.447E-05	6.864E+13	2.463E+15	9.253E+16	3.026E+07
	Std	4.482E-04	8.918E+24	3.153E+41	4.497E-05	7.246E-05	1.115E+14	1.457E+16	2.216E+17	1.529E+08
	Worst	2.693E-03	3.839E+25	9.288E+41	1.775E-04	4.143E-04	2.459E+14	8.743E+16	1.268E+18	9.198E+08
	Median	1.035E-04	3.984E+14	1.811E+40	1.775E-04	6.643E-05	4.803E+11	1.286E+03	1.682E+16	1.596E+06
	Best	3.855E-07	4.969E+11	6.633E+34	7.871E-05	2.050E-05	1.032E+10	1.278E-05	6.469E+13	2.559E+04
	p-value	~	2.927E-13	2.894E-13	3.764E-02	2.055E-02	8.390E-14	2.885E-10	2.927E-13	2.927E-13
	Rank	3	8 (+)	9 (+)	2 (–)	1 (–)	5 (+)	6 (+)	7 (+)	4 (+)
$F_3$	Mean	8.428E+03	8.868E+02	6.622E+04	1.621E-08	8.906E-10	5.848E+04	1.862E-08	3.528E+04	4.287E+01
	Std	3.124E+04	1.086E+03	5.776E+03	2.429E-09	1.074E-09	1.377E+04	1.003E-07	1.450E+04	9.901E+00
	Worst	1.731E+05	4.792E+03	7.582E+04	1.921E-08	4.477E-09	8.389E+04	6.008E-07	8.112E+04	6.545E+01
	Median	2.842E-13	7.570E+02	6.746E+04	1.430E-08	4.970E-10	5.122E+04	2.274E-13	3.258E+04	4.215E+01
	Best	5.684E-14	7.153E-04	4.735E+04	1.430E-08	2.279E-11	5.122E+04	5.684E-14	9.884E+03	2.870E+01
	p-value	~	5.355E-08	1.908E-11	5.617E-06	9.173E-06	1.163E-11	5.293E-01	8.971E-10	8.147E-07
	Rank	6	5 (+)	9 (+)	2 (–)	1 (–)	8 (+)	3 ($\approx$)	7 (+)	4 (–)
$F_4$	Mean	1.944E+00	1.008E+02	8.517E+03	8.514E+01	3.649E+01	1.594E+02	2.562E+01	9.698E+01	1.038E+02
	Std	2.000E+00	3.403E+01	1.189E+03	1.042E+01	3.513E+01	4.583E+01	2.351E+01	2.480E+01	2.201E+01
	Worst	4.333E+00	2.334E+02	1.049E+04	1.091E+02	8.524E+01	1.909E+02	7.177E+01	1.292E+02	1.411E+02
	Median	3.423E-01	9.475E+01	8.670E+03	8.337E+01	4.786E+01	1.909E+02	1.545E+01	9.795E+01	1.131E+02
	Best	5.213E-04	2.113E+01	6.064E+03	7.410E+01	2.657E-04	6.824E+01	2.735E-01	1.903E+01	6.946E+01
	p-value	~	3.040E-13	3.040E-13	1.038E-13	1.408E-03	1.201E-13	2.918E-11	3.040E-13	3.040E-13
	Rank	1	6 (+)	9 (+)	4 (+)	3 (+)	8 (+)	2 (+)	5 (+)	7 (+)
$F_5$	Mean	7.362E+01	1.826E+02	3.790E+02	7.661E+01	2.034E+02	3.055E+02	9.040E+01	1.374E+02	1.951E+02
	Std	4.741E+01	4.616E+01	1.065E+01	5.765E-14	2.663E+01	3.333E+01	1.978E+01	4.812E+01	1.527E+01
	Worst	3.301E+02	2.927E+02	4.014E+02	7.661E+01	2.468E+02	3.333E+02	1.363E+02	2.493E+02	2.412E+02
	Median	6.666E+01	1.780E+02	3.783E+02	7.661E+01	2.084E+02	3.333E+02	9.452E+01	1.249E+02	1.935E+02
	Best	4.079E+01	1.071E+02	3.543E+02	7.661E+01	1.482E+02	2.667E+02	5.870E+01	4.876E+01	1.563E+02
	p-value	~	6.671E-12	3.042E-13	2.462E-03	5.696E-12	4.264E-13	2.725E-05	1.628E-09	5.696E-12
	Rank	1	5 (+)	9 (+)	2 (+)	7 (+)	8 (+)	3 (+)	4 (+)	6 (+)
$F_6$	Mean	1.944E+00	2.492E+01	7.657E+01	3.228E+01	1.584E+01	5.964E+01	1.910E-01	2.757E+01	6.086E+01
	Std	2.591E+00	8.458E+00	5.468E+00	8.791E+00	5.043E+00	2.762E+00	4.765E-01	1.082E+01	4.752E+00
	Worst	1.231E+01	4.112E+01	8.766E+01	4.626E+01	2.493E+01	6.867E+01	2.710E+00	6.039E+01	6.881E+01
	Median	1.141E+00	2.397E+01	7.726E+01	2.691E+01	1.549E+01	5.882E+01	2.767E-02	2.579E+01	6.219E+01
	Best	2.616E-02	8.876E+00	6.204E+01	2.691E+01	6.279E+00	5.882E+01	1.137E-13	9.153E+00	5.161E+01
	p-value	~	3.597E-13	3.044E-13	7.399E-14	1.052E-12	1.706E-14	1.417E-09	3.309E-13	3.044E-13
	Rank	2	4 (+)	9 (+)	6 (+)	3 (+)	7 (+)	1 (–)	5 (+)	8 (+)
$F_7$	Mean	1.098E+02	1.975E+02	6.043E+02	1.486E+02	3.028E+02	5.250E+02	1.134E+02	2.424E+02	4.010E+02
	Std	2.595E+01	5.150E+01	2.843E+01	2.300E+01	7.290E+01	6.435E+01	2.781E+01	7.761E+01	6.029E+01
	Worst	2.141E+02	3.114E+02	6.548E+02	1.788E+02	4.202E+02	5.885E+02	1.688E+02	3.966E+02	5.715E+02
	Median	1.010E+02	1.839E+02	6.117E+02	1.316E+02	3.104E+02	5.250E+02	1.068E+02	2.414E+02	3.924E+02
	Best	7.477E+01	1.098E+02	5.276E+02	1.316E+02	1.494E+02	4.616E+02	6.382E+01	1.198E+02	2.751E+02
	p-value	~	1.460E-11	3.044E-13	1.182E-08	4.618E-13	1.275E-13	5.468E-01	7.224E-12	3.045E-13
	Rank	1	4 (+)	9 (+)	3 (+)	6 (+)	8 (+)	2 ($\approx$)	5 (+)	7 (+)
$F_8$	Mean	7.230E+01	1.886E+02	2.949E+02	1.503E+02	1.594E+02	2.144E+02	9.405E+01	1.391E+02	1.333E+02
	Std	2.056E+01	3.825E+01	1.789E+01	1.225E+01	2.171E+01	4.807E+01	2.483E+01	4.198E+01	2.348E+01
	Worst	1.333E+02	2.711E+02	3.230E+02	1.532E+02	2.000E+02	2.418E+02	1.761E+02	3.199E+02	1.806E+02
	Median	7.114E+01	1.847E+02	2.957E+02	1.532E+02	1.597E+02	2.418E+02	8.805E+01	1.334E+02	1.342E+02
	Best	4.079E+01	1.166E+02	2.580E+02	1.005E+02	9.552E+01	1.323E+02	5.671E+01	8.756E+01	9.883E+01
	p-value	~	3.597E-13	3.044E-13	2.654E-14	5.449E-13	1.405E-13	1.228E-04	6.674E-12	3.830E-12
	Rank	1	7 (+)	9 (+)	5 (+)	6 (+)	8 (+)	2 (+)	4 (+)	3 (+)

**Table 3:** Experimental results of the RSO, DBO, BWO, SSA, AVOA, WOA, ARBBPSO, GTO and HHO for $F_{9}$ - $F_{16}$ with 30 dimension.

Function	Type	RSO	DBO	BWO	SSA	AVOA	WOA	ARBBPSO	GTO	HHO
$F_9$	Mean	5.760E+02	3.350E+03	8.217E+03	1.670E+02	3.709E+03	4.555E+03	1.153E+02	3.698E+03	4.565E+03
	Std	9.079E+02	1.400E+03	8.203E+02	2.829E+02	4.772E+02	2.373E+02	1.664E+02	2.985E+03	4.481E+02
	Worst	3.889E+03	6.727E+03	9.616E+03	1.317E+03	4.556E+03	4.848E+03	7.176E+02	1.091E+04	5.690E+03
	Median	2.348E+02	3.420E+03	8.394E+03	9.936E+01	3.691E+03	4.368E+03	5.325E+01	2.462E+03	4.465E+03
	Best	7.444E+00	6.827E+02	6.200E+03	9.936E+01	2.590E+03	4.368E+03	4.633E+00	8.838E+02	3.710E+03
	p-value	~	2.708E-11	3.046E-13	2.091E-01	7.818E-12	1.116E-13	1.727E-04	1.527E-10	3.913E-13
	Rank	3	4 (+)	9 (+)	2 ($\approx$)	6 (+)	7 (+)	1 (–)	5 (+)	8 (+)
$F_{10}$	Mean	3.215E+03	3.958E+03	6.904E+03	3.785E+03	3.343E+03	5.122E+03	3.271E+03	6.845E+03	4.357E+03
	Std	4.415E+02	6.763E+02	2.512E+02	2.760E+02	5.236E+02	1.259E+02	8.500E+02	1.509E+03	6.378E+02
	Worst	4.129E+03	5.381E+03	7.417E+03	4.555E+03	4.240E+03	5.500E+03	6.154E+03	1.032E+04	5.733E+03
	Median	3.244E+03	3.783E+03	6.944E+03	3.689E+03	3.413E+03	5.043E+03	3.163E+03	7.099E+03	4.387E+03
	Best	2.081E+03	2.813E+03	6.373E+03	3.689E+03	2.350E+03	5.043E+03	2.072E+03	3.564E+03	2.696E+03
	p-value	~	7.012E-07	3.046E-13	9.004E-10	2.625E-01	8.650E-14	6.810E-01	1.345E-12	5.260E-10
	Rank	1	5 (+)	9 (+)	4 (+)	3 ($\approx$)	7 (+)	2 ($\approx$)	8 (+)	6 (+)
$F_{11}$	Mean	4.124E+01	2.608E+02	3.606E+03	1.823E+02	1.397E+02	1.195E+02	5.295E+01	1.164E+02	1.349E+02
	Std	2.303E+01	1.026E+02	6.454E+02	1.537E+01	5.464E+01	3.645E+01	2.136E+01	3.582E+01	3.093E+01
	Worst	9.751E+01	4.560E+02	4.544E+03	1.966E+02	2.918E+02	2.211E+02	1.205E+02	2.055E+02	2.316E+02
	Median	3.831E+01	2.462E+02	3.603E+03	1.966E+02	1.375E+02	1.068E+02	5.196E+01	1.123E+02	1.312E+02
	Best	9.953E+00	5.284E+01	1.859E+03	1.663E+02	4.323E+01	1.068E+02	1.810E+01	4.761E+01	6.778E+01
	p-value	~	6.986E-13	3.046E-13	1.265E-13	2.508E-11	2.248E-14	1.570E-02	1.579E-11	6.986E-13
	Rank	1	8 (+)	9 (+)	7 (+)	6 (+)	4 (+)	2 (+)	3 (+)	5 (+)
$F_{12}$	Mean	4.684E+04	9.718E+06	6.658E+09	7.192E+05	3.658E+05	3.284E+07	1.527E+05	1.474E+06	7.067E+06
	Std	3.288E+04	1.035E+07	1.020E+09	2.628E+05	4.489E+05	2.553E+07	2.199E+05	1.149E+06	2.682E+06
	Worst	1.362E+05	4.156E+07	8.638E+09	1.364E+06	2.425E+06	5.658E+07	1.285E+06	4.900E+06	1.366E+07
	Median	3.643E+04	5.203E+06	6.476E+09	6.152E+05	2.054E+05	5.658E+07	1.050E+05	1.254E+06	6.815E+06
	Best	4.136E+03	1.080E+05	4.397E+09	6.152E+05	4.740E+04	1.373E+06	1.268E+04	4.030E+04	3.187E+06
	p-value	~	4.252E-13	3.046E-13	2.892E-14	5.792E-11	1.734E-13	1.095E-04	3.539E-12	3.046E-13
	Rank	1	7 (+)	9 (+)	4 (+)	3 (+)	8 (+)	2 (+)	5 (+)	6 (+)
$F_{13}$	Mean	2.027E+04	2.963E+05	2.174E+09	6.470E+04	1.765E+04	1.102E+05	1.978E+04	1.206E+04	1.336E+05
	Std	2.488E+04	5.516E+05	8.546E+08	8.630E+03	1.077E+04	3.230E+04	1.827E+04	1.469E+04	3.788E+04
	Worst	8.469E+04	2.561E+06	4.127E+09	7.369E+04	5.340E+04	2.986E+05	7.900E+04	6.221E+04	2.706E+05
	Median	6.407E+03	8.581E+04	1.978E+09	5.665E+04	1.449E+04	1.048E+05	1.728E+04	7.306E+03	1.252E+05
	Best	3.433E+01	9.221E+03	5.630E+08	5.665E+04	5.300E+03	1.048E+05	1.239E+02	7.625E+02	7.309E+04
	p-value	~	2.107E-08	3.046E-13	2.965E-09	7.798E-02	9.133E-15	3.766E-01	6.646E-01	3.600E-13
	Rank	4	8 (+)	9 (+)	5 (+)	2 ($\approx$)	6 (+)	3 ($\approx$)	1 ($\approx$)	7 (+)
$F_{14}$	Mean	6.228E+03	5.009E+04	6.490E+05	1.591E+03	9.359E+03	1.367E+05	5.585E+03	1.131E+04	6.371E+03
	Std	9.788E+03	7.062E+04	3.424E+05	2.146E+03	4.532E+03	1.759E+04	6.625E+03	2.849E+04	2.962E+03
	Worst	4.419E+04	2.731E+05	1.936E+06	5.003E+03	1.881E+04	1.396E+05	3.583E+04	1.514E+05	1.404E+04
	Median	3.270E+03	2.185E+04	6.105E+05	2.785E+02	8.582E+03	1.396E+05	3.857E+03	2.890E+03	5.996E+03
	Best	4.230E+02	5.989E+02	1.658E+05	2.785E+02	1.761E+03	3.407E+04	1.986E+02	2.255E+02	1.530E+03
	p-value	~	3.579E-06	3.046E-13	1.412E-06	1.735E-05	1.103E-14	8.792E-01	7.826E-01	3.731E-03
	Rank	3	7 (+)	9 (+)	1 (–)	5 (+)	8 (+)	2 ($\approx$)	6 ($\approx$)	4 (+)
$F_{15}$	Mean	9.447E+03	4.357E+04	6.677E+07	4.062E+04	1.139E+04	1.583E+05	1.225E+04	8.341E+03	1.420E+04
	Std	9.501E+03	4.600E+04	3.515E+07	1.150E+04	9.988E+03	9.104E+04	1.095E+04	1.280E+04	5.257E+03
	Worst	5.013E+04	2.050E+05	1.419E+08	7.269E+04	4.281E+04	2.629E+05	4.100E+04	5.845E+04	2.775E+04
	Median	7.470E+03	3.358E+04	6.112E+07	3.661E+04	8.489E+03	9.339E+04	8.671E+03	2.534E+03	1.330E+04
	Best	5.044E+01	2.042E+03	1.468E+07	3.661E+04	1.100E+03	2.440E+04	2.929E+01	2.063E+02	4.582E+03
	p-value	~	1.030E-07	3.046E-13	3.922E-13	2.821E-01	2.591E-13	4.207E-01	2.191E-02	8.279E-05
	Rank	2	7 (+)	9 (+)	6 (+)	3 (+)	8 (+)	4 ($\approx$)	1 (–)	5 (+)
$F_{16}$	Mean	7.710E+02	1.254E+03	2.842E+03	5.512E+02	1.435E+03	1.358E+03	7.284E+02	1.614E+03	1.566E+03
	Std	2.266E+02	2.861E+02	2.251E+02	6.137E+00	2.946E+02	2.306E-13	2.253E+02	6.040E+02	2.985E+02
	Worst	1.106E+03	1.830E+03	3.423E+03	5.712E+02	1.921E+03	1.358E+03	1.113E+03	2.797E+03	2.307E+03
	Median	8.272E+02	1.268E+03	2.821E+03	5.493E+02	1.456E+03	1.358E+03	7.464E+02	1.602E+03	1.549E+03
	Best	3.217E+02	6.296E+02	2.441E+03	5.493E+02	7.385E+02	1.358E+03	1.883E+02	1.711E+02	1.051E+03
	p-value	~	3.482E-09	3.046E-13	1.465E-04	6.733E-11	6.415E-15	4.607E-01	1.333E-08	3.913E-13
	Rank	3	4 (+)	9 (+)	1 (+)	6 (+)	5 (+)	2 ($\approx$)	8 (+)	7 (+)

**Table 4:** Experimental results of the RSO, DBO, BWO, SSA, AVOA, WOA, ARBBPSO, GTO and HHO for $F_{17}$ - $F_{24}$ with 30 dimension.

Function	Type	RSO	DBO	BWO	SSA	AVOA	WOA	ARBBPSO	GTO	HHO
$F_{17}$	Mean	3.896E+02	5.738E+02	1.426E+03	2.540E+02	5.269E+02	9.159E+02	2.627E+02	7.697E+02	7.290E+02
	Std	1.472E+02	1.849E+02	2.028E+02	5.382E+01	2.064E+02	1.380E+02	1.152E+02	3.767E+02	1.986E+02
	Worst	6.611E+02	9.930E+02	1.829E+03	3.862E+02	9.353E+02	1.038E+03	4.907E+02	1.518E+03	9.800E+02
	Median	3.869E+02	5.894E+02	1.438E+03	2.327E+02	5.123E+02	1.038E+03	2.548E+02	7.286E+02	7.671E+02
	Best	4.980E+01	2.027E+02	1.017E+03	2.327E+02	1.288E+02	7.638E+02	8.087E+01	9.131E+01	3.182E+02
	p-value	~	4.675E-05	3.046E-13	2.287E-06	2.495E-03	1.234E-13	2.691E-04	4.212E-06	6.409E-09
	Rank	3	5 (+)	9 (+)	1 (–)	4 (+)	8 (+)	2 (–)	7 (+)	6 (+)
$F_{18}$	Mean	2.348E+04	5.819E+05	9.139E+06	3.219E+04	1.896E+05	2.093E+06	2.094E+05	7.580E+05	1.173E+05
	Std	3.359E+04	6.087E+05	5.523E+06	1.676E+04	1.452E+05	1.553E+06	2.248E+05	1.344E+06	3.030E+04
	Worst	1.889E+05	2.455E+06	2.296E+07	1.003E+05	5.104E+05	3.712E+06	1.146E+06	6.231E+06	1.909E+05
	Median	1.473E+04	4.455E+05	8.364E+06	2.818E+04	1.087E+05	6.447E+05	1.182E+05	1.528E+05	1.256E+05
	Best	1.400E+03	1.334E+04	9.397E+05	2.818E+04	3.241E+04	6.447E+05	1.301E+04	3.804E+04	5.606E+04
	p-value	~	2.322E-11	3.046E-13	8.708E-06	9.147E-12	1.265E-13	4.556E-10	4.867E-12	8.457E-12
	Rank	1	6 (+)	9 (+)	2 (+)	4 (+)	8 (+)	5 (+)	7 (+)	3 (+)
$F_{19}$	Mean	7.133E+03	1.346E+05	1.010E+08	1.578E+05	6.090E+03	1.337E+06	7.366E+03	7.708E+03	6.553E+04
	Std	8.113E+03	2.786E+05	5.215E+07	5.102E+04	8.609E+03	7.278E+04	1.093E+04	8.140E+03	6.340E+04
	Worst	4.114E+04	1.604E+06	2.858E+08	2.602E+05	4.201E+04	1.405E+06	4.362E+04	3.243E+04	2.638E+05
	Median	4.050E+03	5.169E+04	9.207E+07	1.331E+05	2.396E+03	1.405E+06	1.616E+03	4.663E+03	4.300E+04
	Best	1.064E+02	5.248E+02	2.025E+07	1.331E+05	2.847E+02	1.262E+06	2.070E+01	2.255E+02	8.658E+03
	p-value	~	4.573E-09	3.046E-13	4.478E-14	1.609E-01	1.265E-13	9.224E-02	8.880E-01	1.644E-10
	Rank	2	6 (+)	9 (+)	7 (+)	1 ($\approx$)	8 (+)	3 ($\approx$)	4 ($\approx$)	5 (+)
$F_{20}$	Mean	4.093E+02	5.093E+02	7.282E+02	3.396E+02	4.452E+02	5.511E+02	1.917E+02	7.555E+02	5.915E+02
	Std	1.938E+02	1.756E+02	8.306E+01	0.000E+00	1.154E+02	4.317E+01	9.641E+01	2.902E+02	1.206E+02
	Worst	8.251E+02	8.797E+02	8.904E+02	3.396E+02	7.619E+02	5.775E+02	4.399E+02	1.232E+03	7.286E+02
	Median	4.310E+02	4.857E+02	7.286E+02	3.396E+02	4.645E+02	5.775E+02	1.925E+02	8.004E+02	6.445E+02
	Best	6.674E+01	1.757E+02	5.644E+02	3.396E+02	2.215E+02	4.825E+02	4.192E+01	2.589E+02	2.675E+02
	p-value	~	3.877E-02	2.373E-10	3.069E-02	4.142E-01	8.241E-04	5.511E-06	1.049E-06	2.347E-05
	Rank	3	5 (+)	8 (+)	2 (–)	4 ($\approx$)	6 (+)	1 (–)	9 (+)	7 (+)
$F_{21}$	Mean	2.752E+02	3.938E+02	5.385E+02	3.420E+02	3.615E+02	5.117E+02	2.940E+02	3.327E+02	4.185E+02
	Std	2.794E+01	3.880E+01	5.068E+01	6.322E+00	2.653E+01	4.276E+01	2.116E+01	5.406E+01	1.074E+01
	Worst	3.591E+02	4.686E+02	5.987E+02	3.430E+02	4.087E+02	5.286E+02	3.456E+02	4.839E+02	4.466E+02
	Median	2.718E+02	3.797E+02	5.548E+02	3.430E+02	3.639E+02	5.286E+02	2.907E+02	3.137E+02	4.202E+02
	Best	2.248E+02	3.197E+02	4.236E+02	3.051E+02	2.853E+02	4.067E+02	2.492E+02	2.587E+02	3.861E+02
	p-value	~	5.923E-13	3.046E-13	3.242E-13	4.495E-12	2.892E-14	6.848E-04	1.165E-07	3.046E-13
	Rank	1	6 (+)	9 (+)	4 (+)	5 (+)	8 (+)	2 (+)	3 (+)	7 (+)
$F_{22}$	Mean	1.804E+03	1.454E+03	5.226E+03	1.000E+02	2.345E+03	5.776E+03	3.927E+03	1.022E+02	4.093E+03
	Std	1.756E+03	1.670E+03	4.329E+02	4.939E-06	2.088E+03	9.440E+02	1.229E+03	2.345E+00	4.244E+02
	Worst	4.100E+03	4.299E+03	5.905E+03	1.000E+02	5.617E+03	6.434E+03	6.676E+03	1.092E+02	5.841E+03
	Median	1.283E+03	1.089E+02	5.260E+03	1.000E+02	3.336E+03	6.434E+03	3.501E+03	1.008E+02	4.052E+03
	Best	1.000E+02	1.000E+02	4.364E+03	1.000E+02	1.000E+02	4.459E+03	2.321E+03	1.002E+02	3.413E+03
	p-value	~	2.339E-01	2.766E-13	6.837E-01	2.030E-03	8.478E-14	2.742E-04	7.650E-01	3.176E-10
	Rank	4	3 ($\approx$)	8 (+)	1 ($\approx$)	5 (+)	9 (+)	6 (+)	2 ($\approx$)	7 (+)
$F_{23}$	Mean	4.356E+02	5.414E+02	8.961E+02	4.439E+02	6.193E+02	6.694E+02	4.533E+02	5.179E+02	7.175E+02
	Std	2.895E+01	5.510E+01	3.504E+01	3.953E+00	5.258E+01	4.115E+01	2.513E+01	6.093E+01	5.941E+01
	Worst	5.056E+02	6.474E+02	9.668E+02	4.445E+02	7.361E+02	8.367E+02	5.089E+02	6.800E+02	9.046E+02
	Median	4.272E+02	5.341E+02	8.932E+02	4.445E+02	6.202E+02	6.595E+02	4.523E+02	5.143E+02	7.121E+02
	Best	3.914E+02	4.492E+02	8.230E+02	4.208E+02	5.148E+02	6.595E+02	3.934E+02	3.928E+02	6.017E+02
	p-value	~	7.226E-12	3.046E-13	1.535E-02	3.046E-13	1.265E-14	3.731E-03	4.272E-09	3.046E-13
	Rank	1	5 (+)	9 (+)	2 (+)	6 (+)	7 (+)	3 (+)	4 (+)	8 (+)
$F_{24}$	Mean	5.080E+02	6.009E+02	9.718E+02	5.018E+02	6.794E+02	6.100E+02	5.331E+02	5.723E+02	1.053E+03
	Std	2.930E+01	5.366E+01	5.573E+01	3.261E+00	7.147E+01	8.624E+00	3.034E+01	5.960E+01	7.886E+01
	Worst	5.915E+02	7.194E+02	1.084E+03	5.034E+02	8.821E+02	6.603E+02	6.018E+02	7.350E+02	1.160E+03
	Median	5.071E+02	5.905E+02	9.732E+02	5.034E+02	6.794E+02	6.085E+02	5.265E+02	5.551E+02	1.093E+03
	Best	4.656E+02	5.337E+02	8.606E+02	4.953E+02	5.520E+02	6.085E+02	4.664E+02	4.875E+02	8.847E+02
	p-value	~	9.893E-12	3.046E-13	6.204E-01	5.453E-13	9.133E-15	4.909E-04	5.509E-08	3.046E-13
	Rank	2	5 (+)	8 (+)	1 ($\approx$)	7 (+)	6 (+)	3 (+)	4 (+)	9 (+)

**Table 5:** Experimental results of the RSO, DBO, BWO, SSA, AVOA, WOA, ARBBPSO, GTO and HHO for $F_{25}$ - $F_{29}$ with 30 dimension.

Function	Type	RSO	DBO	BWO	SSA	AVOA	WOA	ARBBPSO	GTO	HHO
$F_{25}$	Mean	3.812E+02	4.008E+02	1.558E+03	3.869E+02	3.974E+02	4.031E+02	3.807E+02	4.052E+02	3.923E+02
	Std	8.191E+00	2.182E+01	1.233E+02	1.108E-01	1.760E+01	1.319E+01	9.300E+00	1.861E+01	1.334E+01
	Worst	4.144E+02	4.642E+02	1.865E+03	3.871E+02	4.453E+02	4.355E+02	4.208E+02	4.467E+02	4.384E+02
	Median	3.800E+02	3.888E+02	1.593E+03	3.868E+02	3.900E+02	3.979E+02	3.789E+02	3.967E+02	3.879E+02
	Best	3.753E+02	3.836E+02	1.293E+03	3.868E+02	3.836E+02	3.979E+02	3.752E+02	3.859E+02	3.842E+02
	p-value	~	2.708E-11	3.046E-13	2.629E-11	4.980E-11	6.197E-12	1.476E-02	1.579E-11	5.792E-11
	Rank	2	6 (+)	9 (+)	3 (+)	5 (+)	7 (+)	1 (+)	8 (+)	4 (+)
$F_{26}$	Mean	1.834E+03	3.225E+03	6.663E+03	2.113E+03	3.203E+03	3.542E+03	1.655E+03	2.180E+03	4.430E+03
	Std	4.496E+02	8.011E+02	4.165E+02	2.344E+02	9.052E+02	1.600E+02	2.571E+02	1.183E+03	8.439E+02
	Worst	3.106E+03	5.269E+03	7.300E+03	2.216E+03	4.512E+03	4.065E+03	2.382E+03	3.972E+03	5.502E+03
	Median	1.755E+03	3.193E+03	6.725E+03	2.216E+03	3.186E+03	3.494E+03	1.633E+03	2.535E+03	4.561E+03
	Best	1.209E+03	3.025E+02	5.330E+03	1.596E+03	3.000E+02	3.494E+03	1.149E+03	2.012E+02	3.106E+02
	p-value	~	3.156E-11	3.046E-13	3.526E-04	5.650E-10	1.707E-14	1.356E-01	4.459E-03	5.703E-12
	Rank	2	6 (+)	9 (+)	3 (+)	5 (+)	7 (+)	1 (+)	4 (+)	8 (+)
$F_{27}$	Mean	5.000E+02	5.531E+02	9.671E+02	5.340E+02	5.543E+02	5.627E+02	5.000E+02	5.414E+02	5.753E+02
	Std	3.410E-04	3.036E+01	6.049E+01	3.122E+00	1.949E+01	2.784E+01	2.824E-04	2.040E+01	1.881E+01
	Worst	5.000E+02	6.446E+02	1.081E+03	5.358E+02	5.913E+02	5.917E+02	5.000E+02	5.998E+02	6.218E+02
	Median	5.000E+02	5.423E+02	9.660E+02	5.358E+02	5.547E+02	5.367E+02	5.000E+02	5.356E+02	5.770E+02
	Best	5.000E+02	5.096E+02	8.381E+02	5.287E+02	5.184E+02	5.367E+02	5.000E+02	5.134E+02	5.399E+02
	p-value	~	3.046E-13	3.046E-13	6.384E-14	3.046E-13	1.265E-13	3.528E-01	3.046E-13	3.046E-13
	Rank	2	5 (+)	9 (+)	3 (+)	6 (+)	7 (+)	1 ($\approx$)	4 (+)	8 (+)
$F_{28}$	Mean	4.981E+02	4.859E+02	2.804E+03	4.213E+02	3.160E+02	4.471E+02	4.972E+02	4.519E+02	4.300E+02
	Std	4.212E+00	6.125E+01	2.039E+02	4.440E+01	3.879E+01	1.550E+01	4.916E+00	2.076E+01	2.262E+01
	Worst	5.000E+02	6.503E+02	3.233E+03	4.676E+02	4.182E+02	4.566E+02	5.000E+02	5.092E+02	4.772E+02
	Median	5.000E+02	4.705E+02	2.788E+03	3.799E+02	3.000E+02	4.566E+02	5.000E+02	4.527E+02	4.201E+02
	Best	4.882E+02	4.133E+02	2.420E+03	3.799E+02	3.000E+02	4.222E+02	4.882E+02	4.199E+02	3.988E+02
	p-value	~	2.191E-02	3.040E-13	1.262E-13	2.954E-13	7.547E-14	9.730E-01	5.691E-12	3.040E-13
	Rank	8	6 (–)	9 (+)	2 (–)	1 (–)	4 (–)	7 ($\approx$)	5 (–)	3 (–)
$F_{29}$	Mean	7.418E+02	1.097E+03	2.814E+03	1.059E+03	1.079E+03	1.562E+03	5.940E+02	1.575E+03	1.023E+03
	Std	2.024E+02	2.705E+02	3.084E+02	4.434E+01	2.155E+02	1.658E+02	1.573E+02	4.747E+02	4.251E+02
	Worst	1.265E+03	1.727E+03	3.630E+03	1.070E+03	1.446E+03	1.793E+03	1.021E+03	2.675E+03	1.918E+03
	Median	6.875E+02	1.057E+03	2.844E+03	1.070E+03	1.070E+03	1.447E+03	5.990E+02	1.616E+03	7.710E+02
	Best	4.145E+02	7.120E+02	2.033E+03	8.790E+02	6.386E+02	1.447E+03	2.838E+02	7.588E+02	6.499E+02
	p-value	~	8.029E-08	3.046E-13	3.645E-09	7.085E-08	9.417E-14	1.583E-03	2.508E-11	1.465E-03
	Rank	2	6 (+)	9 (+)	4 (+)	5 (+)	7 (+)	1 (–)	8 (+)	3 (+)

**Table 6:** Experimental results of the RSO, DBO, BWO, SSA, AVOA, WOA, ARBBPSO, GTO and HHO for $F_1$ - $F_{2}$ with 100 dimension.

Function	Type	RSO	DBO	BWO	SSA	AVOA	WOA	ARBBPSO	GTO	HHO
$F_1$	Mean	4.126E+03	6.805E+07	2.479E+11	5.994E+03	5.090E+02	5.338E+07	2.431E+03	7.437E+08	2.156E+08
	Std	5.430E+03	9.445E+06	2.476E-04	1.013E+04	1.961E+02	4.437E+06	5.651E+03	2.439E+08	2.314E+06
	Worst	2.379E+02	6.476E+07	2.479E+11	3.055E+02	4.406E+02	4.100E+07	5.056E+02	6.587E+08	2.091E+08
	Median	1.304E+03	6.476E+07	2.479E+11	3.055E+02	4.406E+02	5.492E+07	7.275E+02	6.587E+08	2.164E+08
	Best	1.414E+04	9.439E+07	2.479E+11	2.422E+04	1.056E+03	5.492E+07	3.160E+04	1.424E+09	2.164E+08
	p-value	~	2.972E-13	2.941E-13	1.343E-13	2.948E-12	7.413E-14	6.711E-02	2.972E-13	2.972E-13
	Rank	3	6 (+)	9 (+)	4 (+)	1 (–)	5 (+)	2 (–)	8 (+)	7 (+)
$F_2$	Mean	2.733E+09	2.895E+126	2.108E+164	3.515E+33	4.255E+37	6.971E+134	4.876E+121	9.073E+112	2.115E+70
	Std	1.527E+10	7.572E+126	6.554E+04	1.053E+34	1.431E+38	8.863E+134	2.925E+122	5.051E+113	1.268E+71
	Worst	1.907E+02	1.223E+109	4.821E+159	7.473E+30	1.328E+21	3.083E+110	5.656E+77	7.098E+105	1.434E+62
	Median	2.263E+08	1.922E+121	5.476E+163	2.693E+33	8.000E+25	3.083E+110	1.359E+104	1.184E+108	7.339E+65
	Best	9.179E+10	4.287E+127	1.430E+165	6.449E+34	5.106E+38	1.793E+135	1.755E+123	3.036E+114	7.611E+71
	p-value	~	1.501E-13	1.015E-13	6.303E-14	1.501E-13	6.566E-14	1.689E-13	1.501E-13	1.501E-13
	Rank	1	7 (+)	9 (+)	2 (+)	3 (+)	8 (+)	6 (+)	5 (+)	4 (+)

**Table 7:** Experimental results of the RSO, DBO, BWO, SSA, AVOA, WOA, ARBBPSO, GTO and HHO for $F_3$ - $F_{10}$ with 100 dimension.

Function	Type	RSO	DBO	BWO	SSA	AVOA	WOA	ARBBPSO	GTO	HHO
$F_3$	Mean	3.823E+05	3.163E+05	3.152E+05	6.391E+03	1.745E+04	4.847E+05	1.488E+06	3.869E+05	3.330E+04
	Std	2.679E+05	1.440E+04	7.341E+03	6.321E+02	5.789E+03	1.703E+05	6.339E+05	2.091E+05	6.750E+03
	Worst	8.971E+04	2.702E+05	2.951E+05	5.610E+03	6.192E+03	3.112E+05	8.310E+05	2.677E+05	2.526E+04
	Median	2.920E+05	3.189E+05	3.172E+05	6.888E+03	1.811E+04	6.232E+05	1.403E+06	3.234E+05	3.304E+04
	Best	1.157E+06	3.431E+05	3.359E+05	6.888E+03	2.989E+04	7.821E+05	4.476E+06	1.210E+06	5.118E+04
	p-value	~	1.499E-01	1.050E-01	1.023E-13	2.633E-13	6.184E-04	1.444E-12	1.845E-01	2.633E-13
	Rank	6	5 ($\approx$)	4 ($\approx$)	1 (–)	2 (–)	8 (+)	9 (+)	7 ($\approx$)	3 (–)
$F_4$	Mean	1.262E+02	4.466E+02	7.693E+04	2.531E+02	2.529E+02	6.822E+02	1.552E+02	1.401E+03	4.757E+02
	Std	3.351E+01	8.866E+01	6.553E+03	3.917E+00	5.595E+01	9.329E+01	5.669E+01	2.715E+02	5.530E+01
	Worst	5.254E+01	2.777E+02	5.930E+04	2.500E+02	1.628E+02	5.181E+02	7.155E+01	9.181E+02	3.712E+02
	Median	1.155E+02	4.532E+02	7.736E+04	2.527E+02	2.538E+02	7.392E+02	1.431E+02	1.414E+03	4.627E+02
	Best	1.830E+02	6.206E+02	8.668E+04	2.693E+02	3.619E+02	7.392E+02	2.980E+02	1.914E+03	6.321E+02
	p-value	~	2.972E-13	2.941E-13	1.343E-13	2.948E-12	7.413E-14	6.711E-02	2.972E-13	2.972E-13
	Rank	1	5 (+)	9 (+)	4 (+)	3 (+)	7 (+)	2 ($\approx$)	8 (+)	6 (+)
$F_5$	Mean	5.039E+02	1.132E+03	1.554E+03	7.088E+02	7.133E+02	8.776E+02	1.038E+03	7.905E+02	8.967E+02
	Std	1.308E+02	1.799E+02	2.394E+01	4.755E+01	6.557E+01	1.109E+01	1.193E+02	1.631E+02	3.522E+01
	Worst	2.746E+02	6.820E+02	1.485E+03	6.189E+02	6.278E+02	8.504E+02	8.338E+02	5.526E+02	8.325E+02
	Median	4.726E+02	1.152E+03	1.559E+03	7.353E+02	6.957E+02	8.820E+02	1.030E+03	7.750E+02	9.003E+02
	Best	8.636E+02	1.416E+03	1.591E+03	7.353E+02	8.795E+02	8.820E+02	1.324E+03	1.369E+03	1.017E+03
	p-value	~	4.201E-13	3.008E-13	1.585E-10	9.890E-10	4.511E-14	3.567E-13	2.031E-10	4.565E-13
	Rank	1	8 (+)	9 (+)	2 (+)	3 (+)	5 (+)	7 (+)	4 (+)	6 (+)
$F_6$	Mean	2.849E+01	6.737E+01	1.070E+02	5.593E+01	4.676E+01	7.879E+01	3.371E+01	7.342E+01	7.764E+01
	Std	9.878E+00	9.624E+00	2.271E+00	8.960E-01	3.261E+00	1.253E+00	7.292E+00	1.369E+01	2.759E+00
	Worst	1.274E+01	4.461E+01	1.024E+02	5.440E+01	4.063E+01	7.149E+01	2.305E+01	5.028E+01	7.261E+01
	Median	2.710E+01	6.617E+01	1.070E+02	5.644E+01	4.643E+01	7.900E+01	3.216E+01	7.166E+01	7.769E+01
	Best	5.415E+01	8.784E+01	1.117E+02	5.644E+01	5.479E+01	7.900E+01	5.146E+01	1.029E+02	8.439E+01
	p-value	~	4.597E-13	3.030E-13	6.365E-14	9.748E-11	9.103E-15	7.729E-03	3.581E-13	3.030E-13
	Rank	1	5 (+)	9 (+)	4 (+)	3 (+)	8 (+)	2 (+)	6 (+)	7 (+)
$F_7$	Mean	1.004E+03	1.734E+03	3.037E+03	8.852E+02	2.153E+03	2.573E+03	9.502E+02	1.799E+03	2.821E+03
	Std	3.409E+02	4.775E+02	4.119E+01	1.491E+01	1.395E+02	5.516E+01	1.410E+02	2.912E+02	9.048E+01
	Worst	5.621E+02	1.125E+03	2.949E+03	8.817E+02	1.868E+03	2.560E+03	6.669E+02	1.309E+03	2.541E+03
	Median	8.866E+02	1.627E+03	3.040E+03	8.817E+02	2.177E+03	2.560E+03	9.509E+02	1.787E+03	2.844E+03
	Best	1.794E+03	2.810E+03	3.134E+03	9.458E+02	2.414E+03	2.797E+03	1.262E+03	2.417E+03	3.000E+03
	p-value	~	6.399E-09	3.038E-13	9.858E-01	3.038E-13	1.263E-14	7.826E-01	5.359E-11	3.038E-13
	Rank	3	4 (+)	9 (+)	1 ($\approx$)	6 (+)	7 (+)	2 ($\approx$)	5 (+)	8 (+)
$F_8$	Mean	4.848E+02	1.136E+03	1.697E+03	6.978E+02	9.220E+02	1.140E+03	8.720E+02	8.420E+02	1.068E+03
	Std	1.054E+02	1.519E+02	3.270E+01	1.227E+01	7.717E+01	0.000E+00	1.294E+02	1.828E+02	2.841E+01
	Worst	3.343E+02	8.543E+02	1.609E+03	6.577E+02	7.353E+02	1.140E+03	6.487E+02	6.336E+02	9.713E+02
	Median	4.597E+02	1.134E+03	1.696E+03	7.014E+02	9.472E+02	1.140E+03	8.800E+02	8.198E+02	1.069E+03
	Best	7.681E+02	1.405E+03	1.745E+03	7.014E+02	1.067E+03	1.140E+03	1.123E+03	1.350E+03	1.116E+03
	p-value	~	3.038E-13	3.038E-13	1.509E-11	3.591E-13	6.404E-15	1.140E-12	6.663E-12	3.038E-13
	Rank	1	7 (+)	9 (+)	2 (+)	5 (+)	8 (+)	4 (+)	3 (+)	6 (+)
$F_9$	Mean	1.758E+04	4.005E+04	7.005E+04	2.267E+04	2.242E+04	3.324E+04	3.333E+04	4.951E+04	2.955E+04
	Std	2.319E+04	1.002E+04	2.966E+03	5.468E+02	1.491E+03	4.880E+03	5.913E+03	1.980E+04	3.293E+03
	Worst	1.951E+03	1.956E+04	6.431E+04	2.207E+04	1.936E+04	2.881E+04	2.065E+04	1.984E+04	2.535E+04
	Median	1.237E+04	4.181E+04	7.045E+04	2.281E+04	2.272E+04	2.881E+04	3.376E+04	5.516E+04	2.937E+04
	Best	1.399E+05	5.972E+04	7.494E+04	2.520E+04	2.599E+04	3.859E+04	4.384E+04	9.251E+04	4.272E+04
	p-value	~	1.767E-10	5.691E-12	6.151E-09	1.092E-08	4.752E-11	1.416E-10	8.416E-11	1.053E-10
	Rank	1	7 (+)	9 (+)	3 (+)	2 (+)	5 (+)	6 (+)	8 (+)	4 (+)
$F_{10}$	Mean	1.369E+04	1.741E+04	2.984E+04	1.484E+04	1.561E+04	2.164E+04	2.079E+04	2.886E+04	1.913E+04
	Std	1.201E+03	1.824E+03	4.470E+02	1.437E+03	1.102E+03	1.837E+02	6.694E+03	3.770E+03	1.287E+03
	Worst	1.075E+04	1.455E+04	2.890E+04	1.350E+04	1.324E+04	2.113E+04	1.136E+04	1.883E+04	1.609E+04
	Median	1.385E+04	1.732E+04	2.992E+04	1.350E+04	1.560E+04	2.171E+04	2.208E+04	3.015E+04	1.951E+04
	Best	1.598E+04	2.205E+04	3.057E+04	1.634E+04	1.796E+04	2.171E+04	2.936E+04	3.403E+04	2.135E+04
	p-value	~	8.441E-12	3.040E-13	8.630E-03	2.908E-08	2.246E-14	3.320E-05	3.040E-13	3.040E-13
	Rank	1	4 (+)	9 (+)	2 (+)	3 (+)	7 (+)	6 (+)	8 (+)	5 (+)

**Table 8:** Experimental results of the RSO, DBO, BWO, SSA, AVOA, WOA, ARBBPSO, GTO and HHO for $F_{11}$ - $F_{18}$ with 100 dimension.

Function	Type	RSO	DBO	BWO	SSA	AVOA	WOA	ARBBPSO	GTO	HHO
$F_{11}$	Mean	1.717E+03	9.028E+03	1.870E+05	1.321E+03	1.116E+03	3.755E+03	5.504E+02	1.272E+05	1.875E+03
	Std	5.579E+03	1.175E+04	2.124E+04	9.014E+01	2.373E+02	4.042E+02	1.751E+02	1.265E+05	2.538E+02
	Worst	2.166E+02	2.182E+03	1.427E+05	1.203E+03	6.399E+02	3.628E+03	3.093E+02	2.812E+04	1.391E+03
	Median	6.329E+02	6.604E+03	1.852E+05	1.388E+03	1.122E+03	3.628E+03	5.310E+02	9.313E+04	1.862E+03
	Best	3.411E+04	7.410E+04	2.289E+05	1.388E+03	1.501E+03	5.968E+03	9.307E+02	6.299E+05	2.443E+03
	p-value	~	5.689E-12	3.038E-13	7.903E-08	6.230E-05	1.036E-12	3.375E-02	3.591E-13	2.148E-09
	Rank	4	7 (+)	9 (+)	3 (–)	2 (–)	6 (+)	1 (–)	8 (+)	5 (+)
$F_{12}$	Mean	1.942E+06	3.240E+08	1.639E+11	7.523E+07	1.242E+07	9.825E+08	2.463E+07	4.708E+08	3.262E+08
	Std	1.457E+06	2.682E+08	9.068E+09	0.000E+00	4.766E+06	1.900E+08	1.339E+07	2.266E+08	1.011E+08
	Worst	5.672E+05	3.742E+07	1.399E+11	7.523E+07	5.468E+06	7.001E+08	9.315E+06	1.903E+08	1.392E+08
	Median	1.463E+06	2.945E+08	1.655E+11	7.523E+07	1.143E+07	1.107E+09	2.092E+07	3.977E+08	3.106E+08
	Best	6.157E+06	1.426E+09	1.814E+11	7.523E+07	2.565E+07	1.107E+09	7.568E+07	1.175E+09	7.083E+08
	p-value	~	3.038E-13	3.038E-13	6.404E-15	5.007E-13	8.414E-14	3.041E-13	3.038E-13	3.038E-13
	Rank	1	5 (+)	9 (+)	4 (+)	2 (+)	8 (+)	3 (+)	7 (+)	6 (+)
$F_{13}$	Mean	1.333E+04	5.099E+06	3.595E+10	7.627E+04	4.586E+04	6.739E+04	9.967E+03	3.271E+04	3.325E+06
	Std	1.075E+04	5.601E+06	2.287E+09	1.879E+04	1.160E+04	3.229E+04	1.248E+04	9.525E+03	7.548E+05
	Worst	6.615E+02	1.097E+05	2.990E+10	3.013E+04	2.354E+04	4.901E+04	2.257E+02	1.597E+04	1.725E+06
	Median	1.090E+04	5.079E+06	3.592E+10	8.372E+04	4.317E+04	4.901E+04	5.177E+03	3.145E+04	3.298E+06
	Best	4.804E+04	2.393E+07	4.033E+10	8.372E+04	6.610E+04	1.225E+05	4.537E+04	5.341E+04	4.827E+06
	p-value	~	3.041E-13	3.041E-13	7.116E-14	1.350E-11	6.381E-14	1.475E-02	2.644E-09	3.041E-13
	Rank	2	8 (+)	9 (+)	6 (+)	4 (+)	5 (+)	1 (–)	3 (+)	7 (+)
$F_{14}$	Mean	1.235E+05	3.891E+06	3.558E+07	3.772E+05	2.098E+05	1.509E+06	7.901E+05	6.943E+05	6.035E+05
	Std	7.757E+04	3.787E+06	7.343E+06	5.196E+04	7.322E+04	1.122E+04	4.385E+05	4.373E+05	1.227E+05
	Worst	3.512E+04	2.205E+05	2.223E+07	1.660E+05	7.717E+04	1.507E+06	2.418E+05	1.960E+05	4.028E+05
	Median	1.082E+05	2.669E+06	3.614E+07	3.896E+05	2.100E+05	1.507E+06	6.400E+05	5.828E+05	5.675E+05
	Best	3.991E+05	1.231E+07	4.851E+07	3.896E+05	3.145E+05	1.574E+06	2.164E+06	2.415E+06	1.087E+06
	p-value	~	8.214E-13	3.038E-13	6.690E-13	7.862E-07	9.118E-15	6.420E-13	1.712E-12	3.038E-13
	Rank	1	8 (+)	9 (+)	3 (+)	2 (+)	7 (+)	6 (+)	5 (+)	4 (+)
$F_{15}$	Mean	3.120E+03	4.254E+05	1.687E+10	8.203E+04	2.032E+04	1.110E+05	1.171E+04	6.712E+03	7.467E+05
	Std	2.915E+03	8.441E+05	2.033E+09	4.539E+04	6.690E+03	1.824E+04	1.295E+04	3.279E+03	2.322E+05
	Worst	2.000E+02	2.889E+04	1.233E+10	4.420E+04	6.982E+03	3.683E+04	2.323E+02	1.951E+03	4.369E+05
	Median	2.223E+03	1.457E+05	1.706E+10	4.420E+04	1.929E+04	1.153E+05	8.027E+03	5.898E+03	7.643E+05
	Best	9.861E+03	3.196E+06	2.006E+10	1.350E+05	3.439E+04	1.153E+05	4.797E+04	1.576E+04	1.336E+06
	p-value	~	3.038E-13	3.038E-13	1.182E-13	4.609E-13	1.263E-14	2.191E-02	2.180E-06	3.038E-13
	Rank	1	7 (+)	9 (+)	5 (+)	4 (+)	6 (+)	3 (+)	2 (+)	8 (+)
$F_{16}$	Mean	3.875E+03	6.167E+03	1.730E+04	4.605E+03	4.947E+03	1.059E+04	5.524E+03	5.358E+03	5.610E+03
	Std	6.717E+02	1.046E+03	1.021E+03	2.966E+01	4.950E+02	6.843E+02	1.605E+03	1.319E+03	6.423E+02
	Worst	1.761E+03	3.690E+03	1.467E+04	4.600E+03	3.576E+03	8.913E+03	3.452E+03	3.706E+03	4.385E+03
	Median	3.861E+03	6.130E+03	1.738E+04	4.600E+03	4.945E+03	1.086E+04	5.305E+03	5.205E+03	5.586E+03
	Best	4.980E+03	7.774E+03	1.900E+04	4.778E+03	5.949E+03	1.086E+04	1.124E+04	1.084E+04	7.489E+03
	p-value	~	1.840E-11	3.041E-13	1.780E-08	1.416E-09	2.889E-14	6.847E-09	7.829E-09	1.238E-12
	Rank	1	7 (+)	9 (+)	2 (+)	3 (+)	8 (+)	5 (+)	4 (+)	6 (+)
$F_{17}$	Mean	3.578E+03	5.262E+03	6.766E+05	3.513E+03	4.252E+03	4.544E+03	4.903E+03	4.251E+03	3.912E+03
	Std	1.287E+03	9.381E+02	3.640E+05	3.651E+02	5.346E+02	2.754E+02	1.060E+03	9.086E+02	5.985E+02
	Worst	2.130E+03	3.218E+03	1.238E+05	2.708E+03	3.178E+03	4.498E+03	2.627E+03	2.941E+03	2.858E+03
	Median	3.386E+03	5.252E+03	6.411E+05	3.674E+03	4.265E+03	4.498E+03	5.032E+03	4.059E+03	3.822E+03
	Best	1.029E+04	7.051E+03	1.683E+06	3.674E+03	5.125E+03	6.150E+03	7.503E+03	6.381E+03	5.116E+03
	p-value	~	4.889E-10	3.041E-13	2.352E-01	1.048E-06	2.480E-13	9.085E-08	1.256E-04	5.130E-03
	Rank	2	8 (+)	9 (+)	1 ($\approx$)	5 (+)	6 (+)	7 (+)	4 (+)	3 (+)
$F_{18}$	Mean	3.023E+05	5.852E+06	7.617E+07	6.857E+05	3.669E+05	1.661E+06	3.054E+06	1.844E+06	1.175E+06
	Std	2.795E+05	3.969E+06	1.658E+07	2.945E+05	7.659E+04	1.354E+05	1.575E+06	1.082E+06	3.913E+05
	Worst	1.043E+05	5.261E+05	4.866E+07	4.674E+05	2.403E+05	1.111E+06	7.212E+05	5.053E+05	6.320E+05
	Median	2.020E+05	5.694E+06	7.980E+07	4.674E+05	3.656E+05	1.694E+06	2.753E+06	1.685E+06	1.077E+06
	Best	1.632E+06	1.573E+07	1.076E+08	1.072E+06	6.447E+05	1.694E+06	7.593E+06	4.890E+06	2.894E+06
	p-value	~	5.444E-13	3.041E-13	4.644E-10	7.532E-05	1.522E-14	5.913E-13	2.564E-12	7.215E-12
	Rank	1	8 (+)	9 (+)	3 (+)	2 (+)	5 (+)	7 (+)	6 (+)	4 (+)

**Table 9:** Experimental results of the RSO, DBO, BWO, SSA, AVOA, WOA, ARBBPSO, GTO and HHO for $F_{19}$ - $F_{26}$ with 100 dimension.

Function	Type	RSO	DBO	BWO	SSA	AVOA	WOA	ARBBPSO	GTO	HHO
$F_{19}$	Mean	2.254E+03	2.501E+06	1.534E+10	2.320E+06	1.526E+04	1.572E+07	6.194E+03	1.593E+04	4.039E+06
	Std	3.124E+03	2.555E+06	9.851E+08	5.534E+05	1.280E+04	3.085E+06	1.153E+04	1.551E+04	1.581E+06
	Worst	1.186E+02	5.879E+04	1.343E+10	1.549E+06	1.863E+03	1.384E+07	1.744E+02	1.328E+03	1.671E+06
	Median	1.114E+03	1.396E+06	1.561E+10	2.706E+06	1.029E+04	1.384E+07	1.855E+03	1.083E+04	3.804E+06
	Best	1.709E+04	1.025E+07	1.665E+10	2.706E+06	4.771E+04	2.063E+07	5.043E+04	7.405E+04	8.749E+06
	p-value	~	3.042E-13	3.041E-13	9.408E-14	5.785E-11	7.396E-14	7.424E-02	1.147E-09	3.042E-13
	Rank	1	6 (+)	9 (+)	5 (+)	3 (+)	8 (+)	2 ($\approx$)	4 (+)	7 (+)
$F_{20}$	Mean	2.799E+03	3.813E+03	5.063E+03	4.028E+03	4.002E+03	4.776E+03	4.104E+03	4.983E+03	3.918E+03
	Std	6.534E+02	6.127E+02	2.289E+02	2.306E-12	4.412E+02	2.117E+02	1.118E+03	1.214E+03	4.992E+02
	Worst	1.947E+03	2.577E+03	4.596E+03	4.028E+03	3.097E+03	4.610E+03	2.154E+03	2.807E+03	2.518E+03
	Median	2.719E+03	3.787E+03	5.053E+03	4.028E+03	4.202E+03	4.610E+03	3.873E+03	5.061E+03	3.968E+03
	Best	5.593E+03	5.195E+03	5.465E+03	4.028E+03	4.517E+03	5.265E+03	5.854E+03	7.740E+03	4.944E+03
	p-value	~	4.570E-09	5.698E-12	1.810E-13	1.054E-10	3.375E-12	2.278E-07	9.782E-11	6.995E-10
	Rank	1	2 (+)	9 (+)	5 (+)	4 (+)	7 (+)	6 (+)	8 (+)	3 (+)
$F_{21}$	Mean	7.880E+02	1.453E+03	2.372E+03	9.200E+02	1.261E+03	1.962E+03	1.122E+03	9.931E+02	1.755E+03
	Std	1.286E+02	1.553E+02	7.672E+01	5.900E+01	1.111E+02	9.190E+01	1.649E+02	1.565E+02	1.131E+02
	Worst	6.119E+02	1.168E+03	2.109E+03	6.801E+02	9.812E+02	1.714E+03	7.822E+02	6.501E+02	1.490E+03
	Median	7.778E+02	1.432E+03	2.386E+03	9.341E+02	1.258E+03	2.040E+03	1.123E+03	9.982E+02	1.743E+03
	Best	1.253E+03	1.685E+03	2.483E+03	9.341E+02	1.536E+03	2.040E+03	1.498E+03	1.455E+03	1.992E+03
	p-value	~	4.618E-13	3.045E-13	8.703E-08	1.582E-12	1.304E-13	7.820E-11	2.248E-08	3.045E-13
	Rank	1	6 (+)	9 (+)	2 (+)	5 (+)	8 (+)	4 (+)	3 (+)	7 (+)
$F_{22}$	Mean	1.617E+04	1.876E+04	3.114E+04	1.384E+04	1.600E+04	1.799E+04	2.272E+04	2.730E+04	2.060E+04
	Std	5.519E+03	2.435E+03	5.031E+02	3.928E+02	1.333E+03	3.325E+01	6.565E+03	5.580E+03	1.234E+03
	Worst	1.197E+04	1.464E+04	3.001E+04	1.375E+04	1.411E+04	1.786E+04	1.269E+04	1.597E+04	1.814E+04
	Median	1.428E+04	1.817E+04	3.116E+04	1.375E+04	1.597E+04	1.800E+04	2.454E+04	2.875E+04	2.064E+04
	Best	3.186E+04	2.608E+04	3.205E+04	1.544E+04	1.962E+04	1.800E+04	2.964E+04	3.466E+04	2.482E+04
	p-value	~	9.094E-08	4.240E-10	2.260E-02	4.511E-04	2.037E-09	2.811E-04	4.556E-10	1.423E-08
	Rank	3	5 (+)	9 (+)	1 (–)	2 (–)	4 (+)	7 (+)	8 (+)	6 (+)
$F_{23}$	Mean	1.131E+03	1.801E+03	3.306E+03	1.154E+03	1.468E+03	2.120E+03	1.290E+03	1.460E+03	2.306E+03
	Std	1.194E+02	1.802E+02	1.621E+02	4.612E-13	1.046E+02	0.000E+00	1.210E+02	2.332E+02	1.412E+02
	Worst	9.379E+02	1.445E+03	2.917E+03	1.154E+03	1.249E+03	2.120E+03	1.059E+03	1.106E+03	2.018E+03
	Median	1.126E+03	1.786E+03	3.303E+03	1.154E+03	1.469E+03	2.120E+03	1.288E+03	1.451E+03	2.301E+03
	Best	1.384E+03	2.397E+03	3.636E+03	1.154E+03	1.694E+03	2.120E+03	1.565E+03	2.155E+03	2.574E+03
	p-value	~	3.046E-13	3.046E-13	8.405E-02	1.860E-12	6.415E-15	2.306E-06	3.670E-10	3.046E-13
	Rank	1	6 (+)	9 (+)	2 ($\approx$)	5 (+)	7 (+)	3 (+)	4 (+)	8 (+)
$F_{24}$	Mean	1.630E+03	2.492E+03	5.517E+03	1.623E+03	2.244E+03	3.301E+03	1.928E+03	1.958E+03	3.143E+03
	Std	2.139E+02	3.047E+02	3.277E+02	9.183E+00	1.904E+02	3.228E-12	1.835E+02	1.636E+02	1.736E+02
	Worst	1.315E+03	1.820E+03	4.825E+03	1.603E+03	1.781E+03	3.301E+03	1.547E+03	1.664E+03	2.792E+03
	Median	1.598E+03	2.539E+03	5.512E+03	1.627E+03	2.234E+03	3.301E+03	1.913E+03	1.933E+03	3.173E+03
	Best	2.605E+03	2.946E+03	6.433E+03	1.627E+03	2.671E+03	3.301E+03	2.307E+03	2.386E+03	3.573E+03
	p-value	~	2.017E-12	3.046E-13	1.421E-01	7.226E-12	6.415E-15	5.992E-09	1.644E-10	3.046E-13
	Rank	2	6 (+)	9 (+)	1 ($\approx$)	5 (+)	8 (+)	3 (+)	4 (+)	7 (+)
$F_{25}$	Mean	7.754E+02	2.690E+03	2.207E+04	7.873E+02	7.963E+02	1.170E+03	7.558E+02	1.829E+03	1.002E+03
	Std	6.086E+01	3.884E+03	8.415E+02	4.200E+01	6.309E+01	3.555E+01	5.362E+01	3.107E+02	6.102E+01
	Worst	6.401E+02	8.013E+02	2.033E+04	7.542E+02	6.170E+02	9.987E+02	6.817E+02	1.203E+03	8.572E+02
	Median	7.797E+02	1.022E+03	2.202E+04	7.542E+02	8.277E+02	1.179E+03	7.527E+02	1.800E+03	9.956E+02
	Best	9.091E+02	1.310E+04	2.361E+04	8.392E+02	8.916E+02	1.179E+03	8.946E+02	2.582E+03	1.117E+03
	p-value	~	2.017E-12	3.046E-13	6.341E-01	8.185E-02	1.266E-14	5.924E-02	3.046E-13	4.620E-13
	Rank	2	8 (+)	9 (+)	3 ($\approx$)	4 (+)	6 (+)	1 ($\approx$)	7 (+)	5 (+)
$F_{26}$	Mean	1.204E+04	1.796E+04	4.467E+04	1.114E+04	1.792E+04	2.887E+04	1.459E+04	1.450E+04	2.058E+04
	Std	1.930E+03	3.425E+03	1.376E+03	2.848E+02	2.097E+03	1.107E-11	1.676E+03	2.405E+03	1.650E+03
	Worst	8.250E+03	1.087E+04	4.108E+04	1.044E+04	1.355E+04	2.887E+04	1.094E+04	1.157E+04	1.710E+04
	Median	1.186E+04	1.876E+04	4.483E+04	1.125E+04	1.792E+04	2.887E+04	1.454E+04	1.391E+04	2.028E+04
	Best	1.787E+04	2.402E+04	4.700E+04	1.125E+04	2.270E+04	2.887E+04	1.815E+04	2.219E+04	2.532E+04
	p-value	~	4.893E-09	3.046E-13	1.389E-02	2.783E-12	6.415E-15	4.140E-07	5.511E-06	3.600E-13
	Rank	2	6 (+)	9 (+)	1 (+)	5 (+)	8 (+)	4 (+)	3 (+)	7 (+)

**Table 10:** Experimental results of the RSO, DBO, BWO, SSA, AVOA, WOA, ARBBPSO, GTO and HHO for $F_{27}$ - $F_{29}$ with 100 dimension.

Function	Type	RSO	DBO	BWO	SSA	AVOA	WOA	ARBBPSO	GTO	HHO
$F_{27}$	Mean	5.000E+02	1.192E+03	7.324E+03	7.977E+02	1.191E+03	2.159E+03	5.000E+02	1.010E+03	1.292E+03
	Std	4.150E-04	2.088E+02	6.724E+02	3.459E-13	1.830E+02	1.845E-12	3.723E-04	1.233E+02	1.434E+02
	Worst	5.000E+02	8.590E+02	5.854E+03	7.977E+02	9.335E+02	2.159E+03	5.000E+02	7.807E+02	9.657E+02
	Median	5.000E+02	1.154E+03	7.370E+03	7.977E+02	1.158E+03	2.159E+03	5.000E+02	1.003E+03	1.278E+03
	Best	5.000E+02	1.727E+03	8.365E+03	7.977E+02	1.850E+03	2.159E+03	5.000E+02	1.390E+03	1.606E+03
	p-value	~	3.046E-13	3.046E-13	6.415E-15	3.046E-13	6.415E-15	1.770E-10	3.046E-13	3.046E-13
	Rank	1	6 (+)	9 (+)	3 (+)	5 (+)	8 (+)	2 (+)	4 (+)	7 (+)
$F_{28}$	Mean	5.000E+02	1.050E+04	2.247E+04	5.805E+02	5.611E+02	9.334E+02	5.000E+02	1.905E+03	7.628E+02
	Std	4.778E-04	7.843E+03	5.883E+02	8.306E+00	3.614E+01	1.667E+01	3.972E-04	3.778E+02	5.262E+01
	Worst	5.000E+02	6.625E+02	2.090E+04	5.663E+02	4.989E+02	8.871E+02	5.000E+02	1.290E+03	6.271E+02
	Median	5.000E+02	1.313E+04	2.253E+04	5.852E+02	5.513E+02	9.390E+02	5.000E+02	1.828E+03	7.660E+02
	Best	5.000E+02	2.215E+04	2.333E+04	5.852E+02	6.497E+02	9.475E+02	5.000E+02	2.692E+03	8.457E+02
	p-value	~	3.046E-13	3.046E-13	6.384E-14	1.232E-09	2.907E-14	8.956E-09	3.046E-13	3.046E-13
	Rank	1	8 (+)	9 (+)	4 (+)	3 (+)	6 (+)	2 (+)	7 (+)	5 (+)
$F_{29}$	Mean	3.237E+03	6.243E+03	1.319E+05	5.877E+03	4.877E+03	1.009E+04	4.225E+03	6.040E+03	5.635E+03
	Std	6.894E+02	1.232E+03	6.259E+04	2.137E+02	6.134E+02	3.179E+02	9.061E+02	1.078E+03	5.904E+02
	Worst	1.726E+03	3.819E+03	2.568E+04	5.178E+03	3.972E+03	9.542E+03	2.861E+03	4.313E+03	4.311E+03
	Median	3.397E+03	6.290E+03	1.177E+05	5.941E+03	4.740E+03	1.027E+04	4.021E+03	5.831E+03	5.659E+03
	Best	4.590E+03	9.178E+03	2.614E+05	5.941E+03	6.481E+03	1.027E+04	6.575E+03	9.224E+03	6.883E+03
	p-value	~	5.453E-13	3.046E-13	1.707E-14	2.187E-12	6.384E-14	1.038E-05	3.312E-13	3.312E-13
	Rank	1	7 (+)	9 (+)	5 (+)	3 (+)	8 (+)	2 (+)	6 (+)	4 (+)

**Figure 2:**
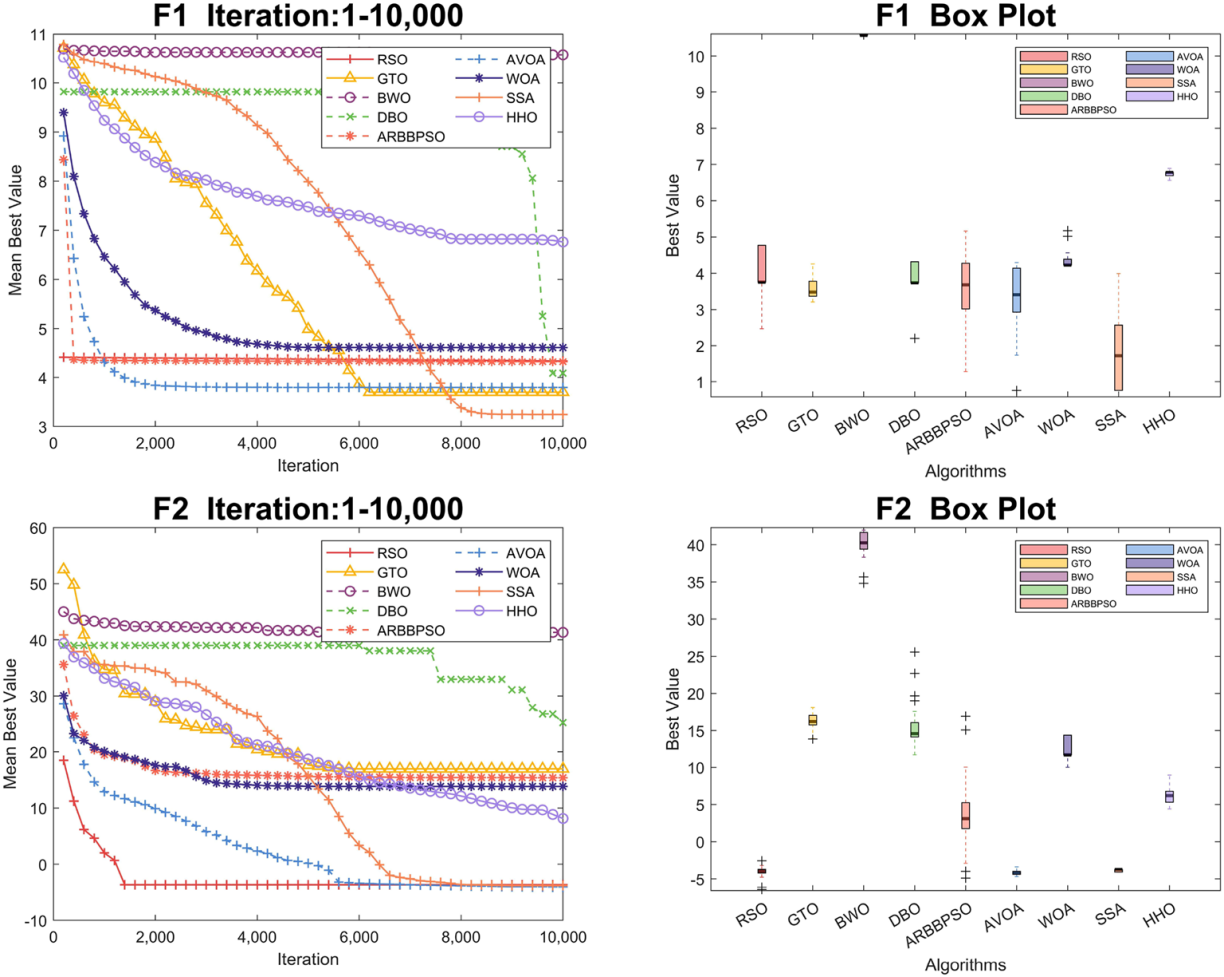
Convergence figures and box plots of RSO, DBO, BWO, SSA, AVOA, WOA, ARBBPSO, GTO and HHO in CEC2017 *F*1 and *F*2 with 30 dimension. The vertical axis of these figures represents the logarithmic transformation of the experimental results.

**Figure 3:**
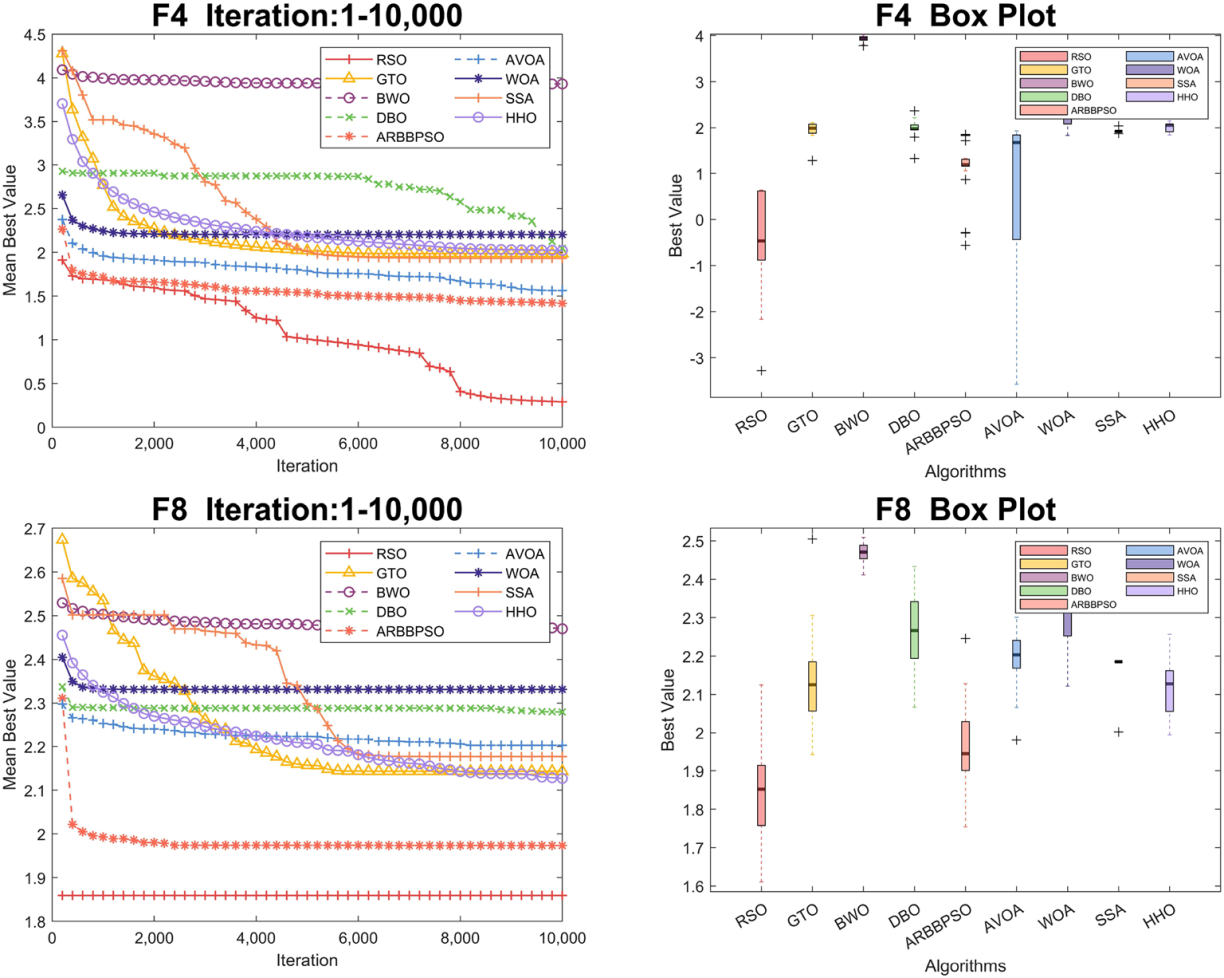
Convergence figures and box plots of RSO, DBO, BWO, SSA, AVOA, WOA, ARBBPSO, GTO and HHO in CEC2017 *F*7 and *F*8 with 30 dimension. The vertical axis of these figures represents the logarithmic transformation of the experimental results.

**Figure 4:**
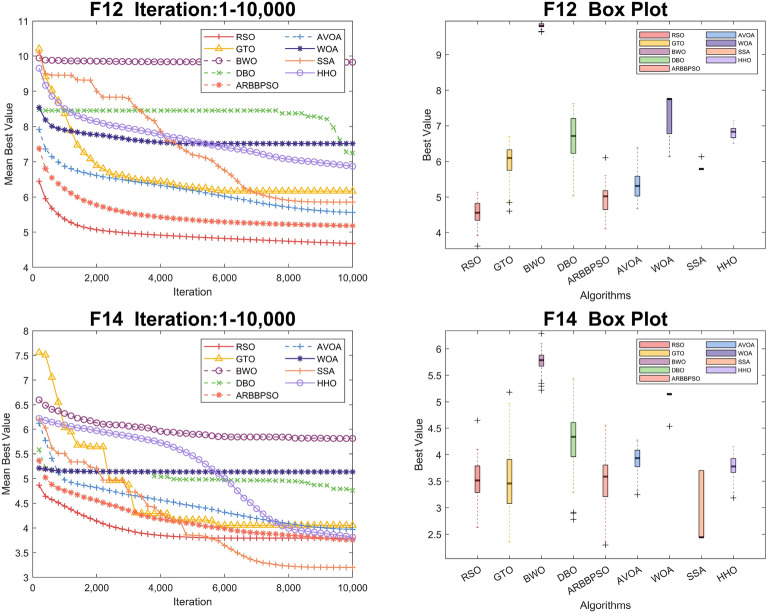
Convergence figures and box plots of RSO, DBO, BWO, SSA, AVOA, WOA, ARBBPSO, GTO and HHO in CEC2017 *F*10 and *F*17 with 30 dimension. The vertical axis of these figures represents the logarithmic transformation of the experimental results.

**Figure 5:**
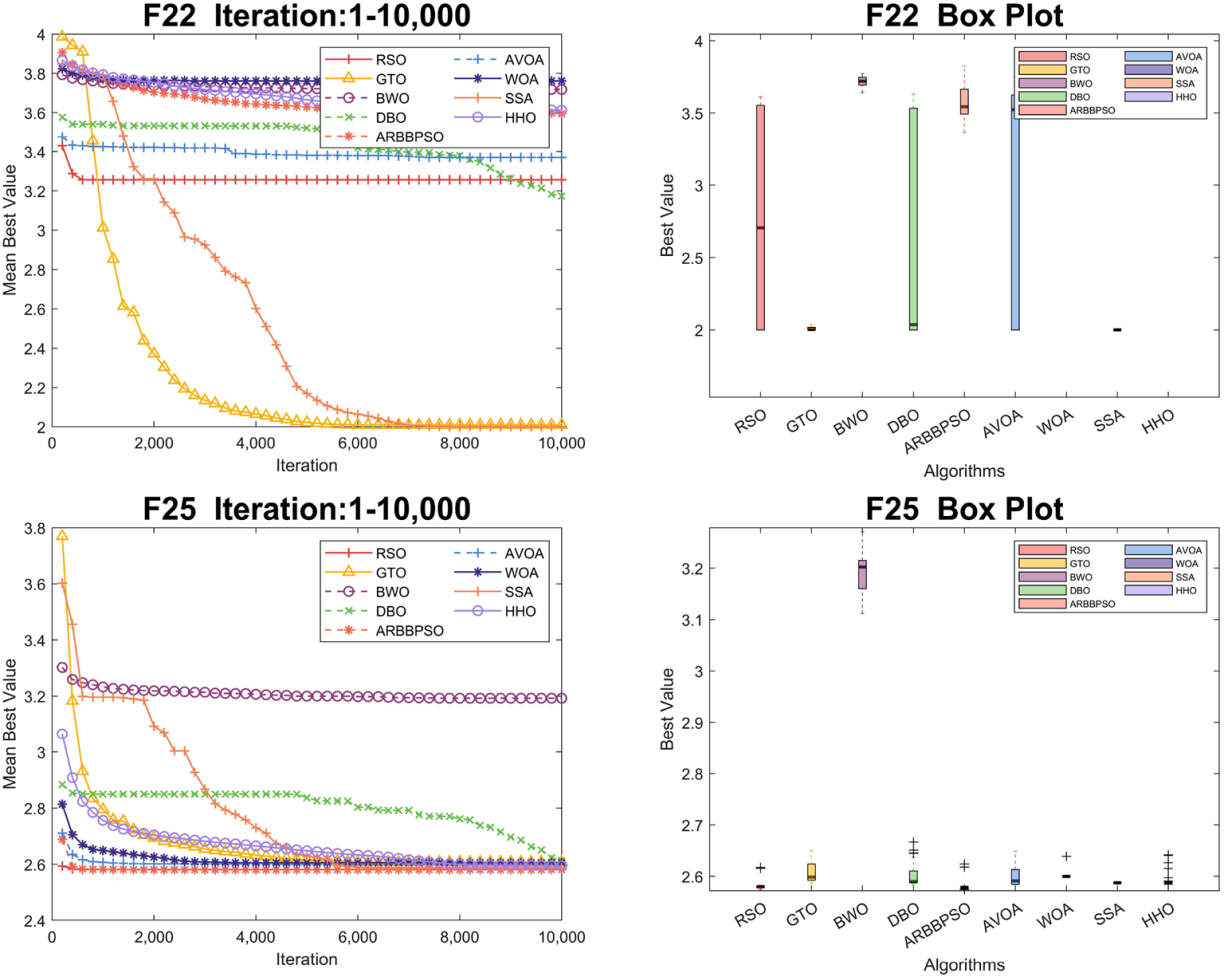
Convergence figures and box plots of RSO, DBO, BWO, SSA, AVOA, WOA, ARBBPSO, GTO and HHO in CEC2017 *F*21 and *F*23 with 30 dimension. The vertical axis of these figures represents the logarithmic transformation of the experimental results.

**Figure 6:**
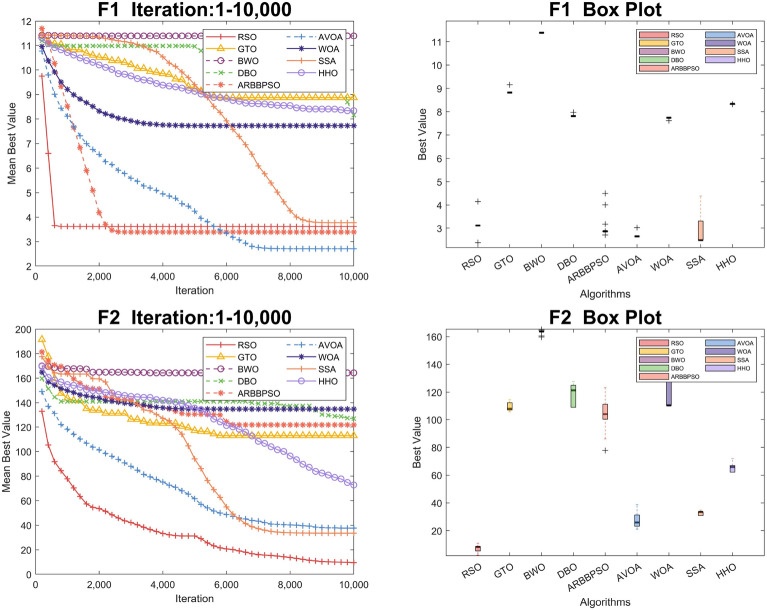
Convergence figures and box plots of RSO, DBO, BWO, SSA, AVOA, WOA, ARBBPSO, GTO and HHO in CEC2017 *F*1 and *F*2 with 100 dimension. The vertical axis of these figures represents the logarithmic transformation of the experimental results.

**Figure 7:**
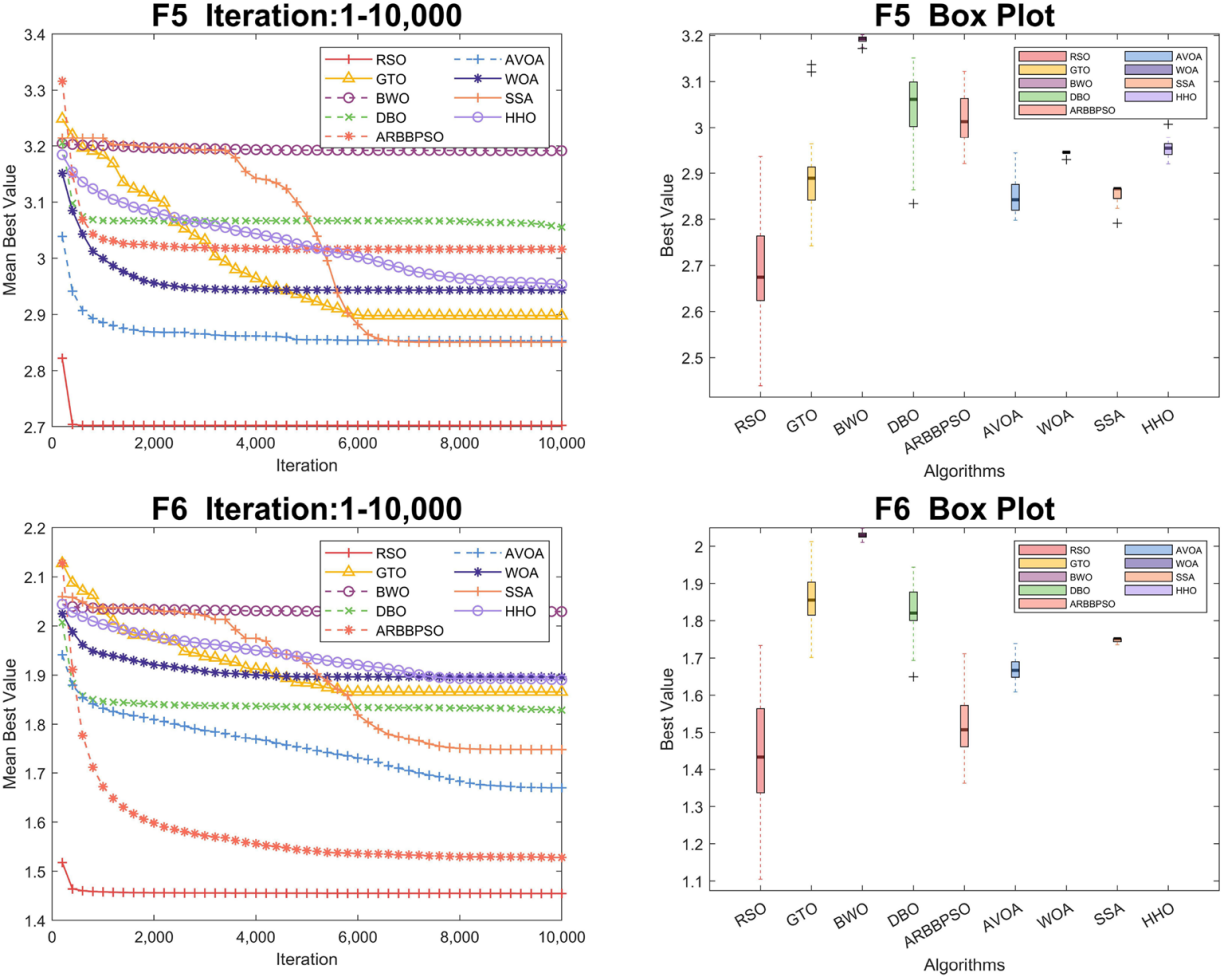
Convergence figures and box plots of RSO, DBO, BWO, SSA, AVOA, WOA, ARBBPSO, GTO and HHO in CEC2017 *F*5 and *F*6 with 100 dimension. The vertical axis of these figures represents the logarithmic transformation of the experimental results.

**Figure 8:**
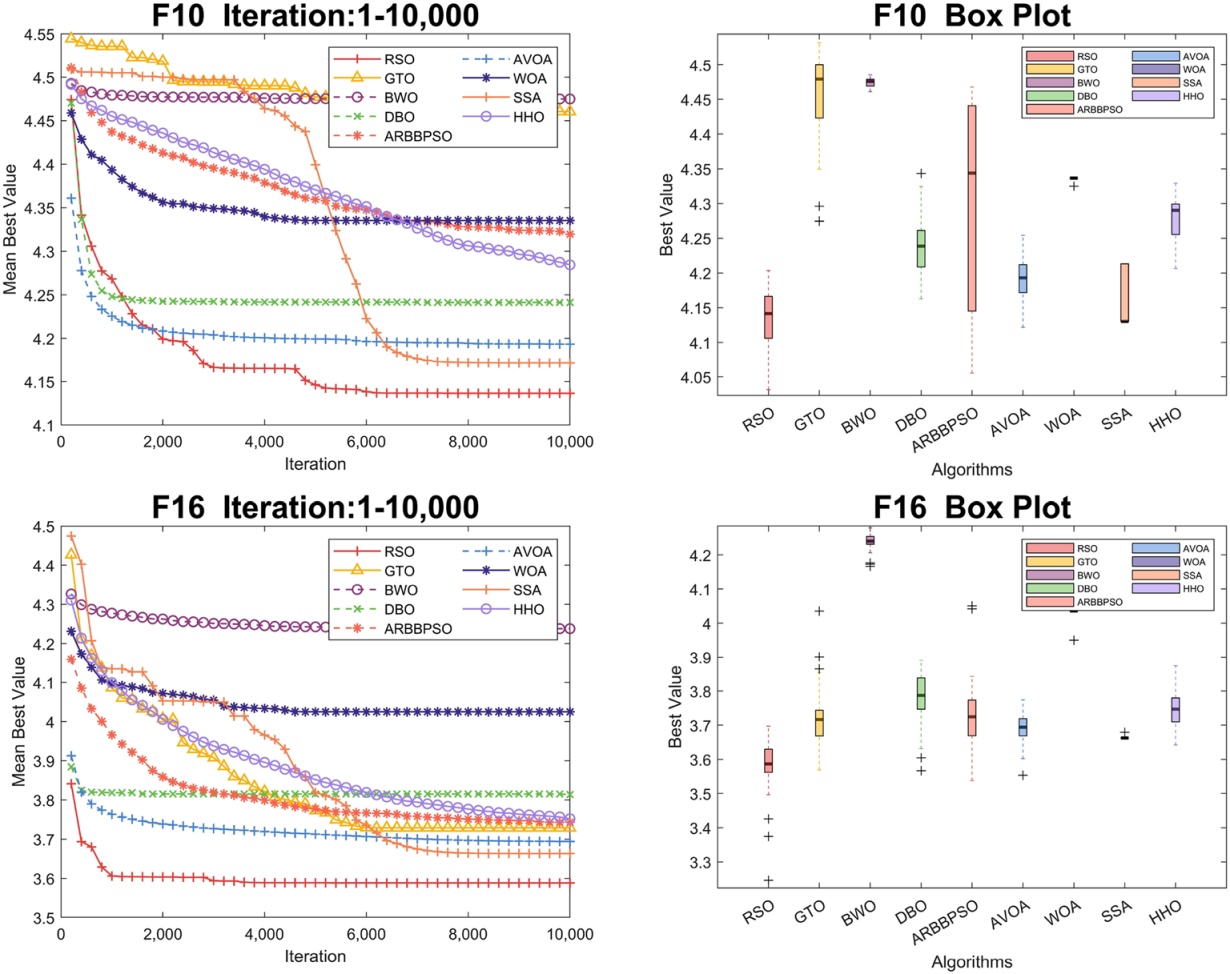
Convergence figures and box plots of RSO, DBO, BWO, SSA, AVOA, WOA, ARBBPSO, GTO and HHO in CEC2017 *F*10 and *F*16 with 100 dimension. The vertical axis of these figures represents the logarithmic transformation of the experimental results.

**Figure 9:**
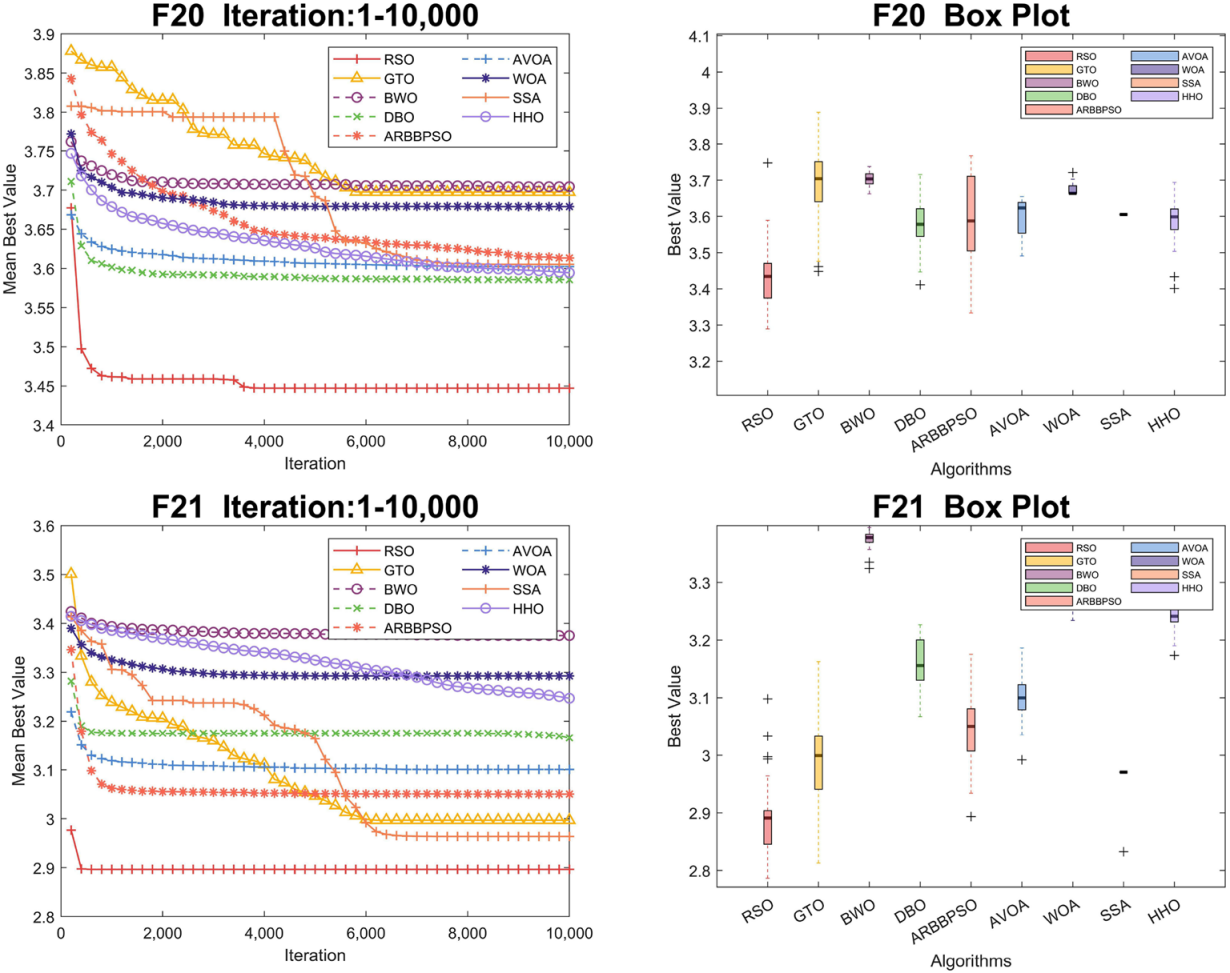
Convergence figures and box plots of RSO, DBO, BWO, SSA, AVOA, WOA, ARBBPSO, GTO and HHO in CEC2017 *F*20 and *F*21 with 100 dimension. The vertical axis of these figures represents the logarithmic transformation of the experimental results.

To visualize the iteration process of the algorithm in 30 and 100 dimension, we selected two test functions from each of the four function types to obtain the convergence curves and box plots, as shown in Figs. 2, 3, 4, 5, 6, 7, 8 and 9. Additionally, we applied a logarithmic transformation to ME used in the convergence figures and box plots to enhance the clarity of these figures.

From the convergence curves of these algorithms in 30 dimension, it is notable that RSO has a significant advantage in local search ability in $F_{2}$ and $F_{22}$. This advantage is attributed to the mimicry strategy, which helps individuals update positions by communicating with individuals of other groups, significantly enhancing the local search capability. In $F_{12}$ and $F_{14}$, RSO continuously updated the best value throughout the iteration process, demonstrating its excellent global search capability. This is because the vertical migration strategy assists individuals in updating their positions based on the optimal positions of the swarm, thus strengthening global search capability. Due to the concerted search strategy, RSO individuals can simultaneously consider the optimal positions of the swarm while updating their positions by combining the individual positions of other groups. This enables RSO to exhibit an outstanding ability to escape local optima in $F_{4}$. Although RSO was trapped in local optima in $F_{8}$ and $F_{25}$, the upper quartiles of RSO in box plots were among the smallest. This result indicates that RSO is accurate in these two functions.

Regarding the iteration process in 100 dimension, the three search strategies also make a significant difference. It is noteworthy that RSO performed better in Unimodal Functions compared to its performance in 30 dimension. In particular, RSO shows the ability to escape local optima during the iteration process in $F_2$. Although RSO converged early in $F_{5}$, $F_{6}$, and $F_{21}$, the mean best values of RSO were consistently lower than those of other algorithms. In $F_{10}$, $F_{16}$, and $F_{20}$, RSO demonstrates the ability to escape local optima due to its strong global and local search capabilities. Additionally, it converged to the lowest mean best value, validating the high accuracy of RSO in these functions.

We further investigated the experimental results using the Wilcoxon signed-rank test and Friedman test. The test results are shown in Tables 11 and 12, where ’+’ denotes that RSO performs better than other algorithms, ’-’ denotes that RSO performs worse than other algorithms, and ’$\approx$’ denotes that RSO performs equally well as other algorithms. “Mean Rank” denotes the average ranking following 36 independent runs, and “Rank” denotes the overall final ranking.

Results in Table 11 indicate that RSO outperformed the control group in up to 29 of the 29 functions with 30 dimension. Furthermore, RSO significantly outperformed other algorithms, except ARBBPSO, in Simple Multimodal, Hybrid, and Composite Functions. However, the performance of RSO in Unimodal Functions was weaker compared to its performance in other types of functions, particularly underperforming SSA and AVOA.

Table 12 shows the performance analysis of RSO in CEC2017 with 100 dimension. RSO outperformed the control group in at least 20 out of the 29 functions. Specifically, compared to DBO, BWO, WOA, GTO, and HHO, RSO excelled in all functions of Unimodal, Hybrid, and Composite Functions. In Multimodal Functions, RSO performed significantly better than the control group in almost all functions, but performed significantly worse than SSA, AVOA, and HHO in only one function.

In the experimental results for both 30 and 100 dimension, the Mean Rank of RSO ranked first, surpassing the second-ranked algorithms by 7.69% and 42.85%, respectively. Although RSO performed well in both 30 and 100 dimension, its performance advantage in 100 dimension was significantly higher than in 30 dimension. This indicates that RSO is more effective at solving high-dimensional optimization problems than low-dimensional ones.

**Table 11:** The result of statistical test in CEC2017 with 30 dimension.

Algorithms	Unimodal	Multimodal	Hybrid	Composite	Sum	Mean rank	Rank
	Functions (+/$\approx$/−)	Functions (+/$\approx$/−)	Functions (+/$\approx$/−)	Functions (+/$\approx$/−)	+/$\approx$/−		
RSO	~	~	~	~	~	2.4828	1
DBO	1/1/0	6/0/1	10/0/0	8/1/1	25/2/2	5.6207	6
BWO	2/0/0	7/0/0	10/0/0	10/0/0	29/0/0	8.8966	9
SSA	0/0/2	5/1/1	7/0/3	6/2/2	18/3/8	3.1034	3
AVOA	0/0/2	6/0/1	6/4/0	8/1/1	20/5/4	4.2069	4
WOA	2/0/0	7/0/0	10/0/0	9/0/1	28/0/1	7.0345	8
ARBBPSO	1/1/0	3/2/2	3/6/1	4/3/3	11/12/6	2.6897	2
GTO	1/0/1	7/0/0	6/3/1	8/1/1	22/4/3	5.0000	5
HHO	2/0/0	6/0/1	10/0/0	9/0/1	27/0/2	5.9655	7

**Table 12:** The result of statistical test in CEC2017 with 100 dimension.

Algorithms	Unimodal	Multimodal	Hybrid	Composite	Sum	Mean rank	Rank
	Functions (+/$\approx$/−)	Functions +/$\approx$/−)	Functions (+/$\approx$/−)	Functions (+/$\approx$/−)	+/$\approx$/−		
RSO	~	~	~	~	~	1.6552	1
DBO	2/0/0	6/1/0	10/0/0	10/0/0	28/1/0	6.2759	7
BWO	2/0/0	6/1/0	10/0/0	10/0/0	28/1/0	8.8276	9
SSA	2/0/0	5/1/1	8/1/1	5/3/2	20/5/4	2.8966	2
AVOA	1/0/1	6/0/1	9/0/1	8/1/1	24/1/4	3.4138	3
WOA	2/0/0	7/0/0	10/0/0	10/0/0	29/0/0	6.7931	8
ARBBPSO	1/0/1	5/2/0	7/1/2	9/1/0	22/4/3	3.9655	4
GTO	2/0/0	6/1/0	10/0/0	10/0/0	28/1/0	5.4828	5
HHO	2/0/0	6/0/1	10/0/0	10/0/0	28/0/1	5.6897	6

## Engineering design problems

In this section, we simulated RSO on some engineering design problems^44^ including 10-Bar Truss Design (10-BTD), Gear Train Design (GTD), Three-Bar Truss Design (3-BTD) to demonstrate its efficiency. These problems incorporate the satisfaction of constraints and the search for optimal solution. The effectiveness of optimization algorithms is often significantly affected by the constraints and a limited solution space. Therefore, an added mechanism called Constraint Handling Technique (CHT) is required to address these challenges. This technique ensures the final solution meets constraints by imposing penalties on solutions that do not satisfy them. To represent the fairness of the experiment, we ran each algorithm individually 36 times on all problems with the max number of iteration 500 and population size 50.

### 10-bar truss design

The 10-BTD is a significant engineering design problem with the objective on weight minimization of the truss structure while satisfying the frequency constraints. The details of the problem are as follows:

Minimize:

6 $$f({\bar{x}}) = \sum \limits _{i=1}^{10}{{L_i(x_i)}\rho _i A_i}$$

Subject to:

7 $$\begin{aligned} g_1({\bar{x}})&=\frac{7}{\omega _1({\bar{x}})}-1\le 0, \\ g_2({\bar{x}})&=\frac{15}{\omega _2({\bar{x}})}-1\le 0, \\ g_3({\bar{x}})&=\frac{20}{\omega _3({\bar{x}})}-1\le 0. \\ \end{aligned}$$

With bounds:

8 $$\begin{aligned}&6.45\times 10^{-5}\le A_i \le 5 \times 10^{-3}, i=1,2,\ldots ,10. \\&{\bar{x}}=[A_1,A_2,\ldots ,A_{10}],\rho =2770.\\ \end{aligned}$$

**Table 13:** Results analysis of 10-BTD, Part 1.

	$x_1$	$x_2$	$x_3$	$x_4$	$x_5$	*x*6	$x_7$	$x_8$	$x_9$	$x_{10}$	cost
RSO	**3.3828E-03**	**1.4758E-03**	**3.6725E-03**	**1.4262E-03**	**6.4502E-05**	**4.5776E-04**	**2.4450E-03**	**2.3391E-03**	**1.1955E-03**	**1.2612E-03**	**5.2662E+02**
GTO	3.5678E-03	1.3691E-03	3.5895E-03	1.5270E-03	6.4595E-05	4.6817E-04	2.2075E-03	2.5955E-03	1.1978E-03	1.1914E-03	5.3533E+02
BWO	3.7737E-03	1.8979E-03	3.2435E-03	1.6901E-03	6.4500E-05	4.9818E-04	2.4646E-03	2.1503E-03	1.6261E-03	1.0257E-03	5.5588E+02
DBO	3.5147E-03	1.3798E-03	3.5745E-03	1.5902E-03	6.4500E-05	4.5786E-04	2.1391E-03	2.5816E-03	1.2677E-03	1.1943E-03	5.4272E+02
ARBBPSO	3.2868E-03	1.5304E-03	3.7654E-03	1.5097E-03	6.4500E-05	4.5102E-04	2.3613E-03	2.2996E-03	1.1547E-03	1.3578E-03	5.3108E+02
AVOA	3.4918E-03	1.5020E-03	3.4623E-03	1.6184E-03	6.4500E-05	4.6492E-04	2.2238E-03	2.3673E-03	1.3715E-03	1.2241E-03	5.3712E+02
WOA	4.2977E-03	1.9830E-03	4.1896E-03	1.8099E-03	5.7974E-04	4.0600E-04	2.8678E-03	1.3802E-03	7.6989E-04	1.4035E-03	6.6741E+02
SSA	3.6147E-03	1.4354E-03	3.4136E-03	1.2566E-03	6.4500E-05	4.6999E-04	2.6987E-03	2.3018E-03	1.1396E-03	1.3062E-03	5.3302E+02
HHO	3.1121E-03	1.9096E-03	2.8714E-03	1.3130E-03	1.2138E-03	1.9554E-04	2.1363E-03	2.6682E-03	1.8730E-03	2.1947E-03	6.8513E+02

Significant values are in bold.

**Table 14:** Results analysis of 10-BTD, Part 2.

	Worst	Mean	Best	Std
RSO	**5.3296E+02**	**5.2480E+02**	**5.2662E+02**	**2.2839E+00**
GTO	5.5236E+02	5.2576E+02	5.3533E+02	5.8070E+00
BWO	5.7826E+02	5.4316E+02	5.5588E+02	8.4406E+00
DBO	5.9015E+02	5.2531E+02	5.4272E+02	1.6380E+01
ARBBPSO	5.3807E+02	5.2564E+02	5.3108E+02	3.0046E+00
AVOA	6.0297E+02	5.2602E+02	5.3712E+02	1.2774E+01
WOA	7.6871E+02	5.6603E+02	6.6741E+02	4.8853E+01
SSA	5.4073E+02	5.2647E+02	5.3302E+02	4.1307E+00
HHO	7.6103E+02	5.8668E+02	6.8513E+02	4.7818E+01

Significant values are in bold.

**Figure 10:**
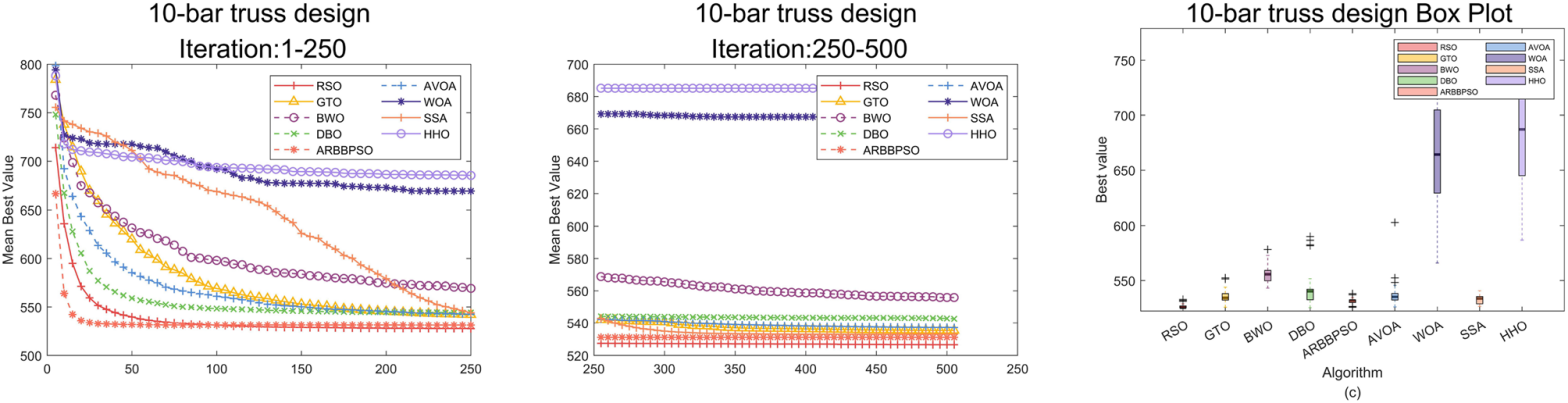
Convergence curves and error bars of RSO and the control group algorithms on 10-BTD.

Tables 13 and 14 present the results of RSO and other algorithms on 10-BTD. In the Table 13, RSO receives the best solution with ${\bar{x}}$ =(3.3828E-03, 1.4758E-03, 3.6725E-03, 1.4262E-03, 6.4502E-05, 4.5776E-04, 2.4450E-03, 2.3391E-03, 1.1955E-03, 1.2612E-03) and optimal value 5.2662E+02. From the Table 14, it is obvious that RSO demonstrates superior values across Best, Mean, Worst, and Std compared to other algorithms. To visualize the iteration process of these algorithms on 10-BTD, the convergence curves and error bars of RSO and the control group are shown in Fig. 10. Although RSO converged early in the iteration process, the distance between upper and lower quartile in the box plot was the smallest. This indicates that while RSO may have been trapped into a local optimum, it exhibits a robust stability when solving 10-BTD problem.

### Gear train design

The GTD is a critical engineering problem focusing on the gear ratio minimization in a compound gear train arrangement. The details of the problem are as follows:

Minimize:

9 $$f({\bar{x}}) = (i_{trg}-i_{tot})^2 = \left( \frac{1}{6.931}-\frac{x_1 x_2}{x_3 x_4}\right) ^2$$

Subject to:

10 $$\begin{aligned} g_{1-4}({\bar{x}})&=12-x_i\le 0, \\ g_{5-8}({\bar{x}})&=(60-{\bar{x}})\le 0, \\ \end{aligned}$$

With bounds:

11 $$12 \le x_i \le 60, i=1,2,\ldots ,4.$$

**Table 15:** Results analysis of GTD, Part 1.

	$x_1$	$x_2$	$x_3$	$x_4$	cost
RSO	**1.4669E+01**	**1.8044E+01**	**3.8371E+01**	**4.7807E+01**	**0.0000E+00**
GTO	**1.5813E+01**	**1.2803E+01**	**3.6145E+01**	**3.8821E+01**	**0.0000E+00**
BWO	1.4289E+01	2.6502E+01	5.8459E+01	4.4898E+01	1.1975E-10
DBO	1.7267E+01	1.2000E+01	3.0961E+01	4.6385E+01	1.6049E-22
ARBBPSO	1.9235E+01	1.2789E+01	3.7874E+01	4.5017E+01	3.7384E-21
AVOA	1.2465E+01	3.5991E+01	5.8244E+01	5.3387E+01	1.1021E-32
WOA	1.4719E+01	3.2147E+01	5.9757E+01	5.4880E+01	4.3226E-33
SSA	1.2000E+01	3.8824E+01	5.3820E+01	5.9997E+01	5.4617E-20
HHO	**1.9874E+01**	**2.1403E+01**	**5.5772E+01**	**5.2861E+01**	**0.0000E+00**

Significant values are in bold.

**Table 16:** Results analysis of GTD, Part 2.

	Worst	Mean	Best	Std
RSO	**0.0000E+00**	**0.0000E+00**	**0.0000E+00**	**0.0000E+00**
GTO	**0.0000E+00**	**0.0000E+00**	**0.0000E+00**	**0.0000E+00**
BWO	8.2624E-10	2.9260E-16	1.1975E-10	2.1456E-10
DBO	5.7777E-21	0.0000E+00	1.6049E-22	9.6295E-22
ARBBPSO	7.5920E-20	0.0000E+00	3.7384E-21	1.3752E-20
AVOA	2.2264E-31	0.0000E+00	1.1021E-32	4.2294E-32
WOA	7.7037E-32	0.0000E+00	4.3226E-33	1.7887E-32
SSA	3.3999E-19	2.8827E-22	5.4617E-20	8.7540E-20
HHO	**0.0000E+00**	**0.0000E+00**	**0.0000E+00**	**0.0000E+00**

Significant values are in bold.

**Figure 11:**
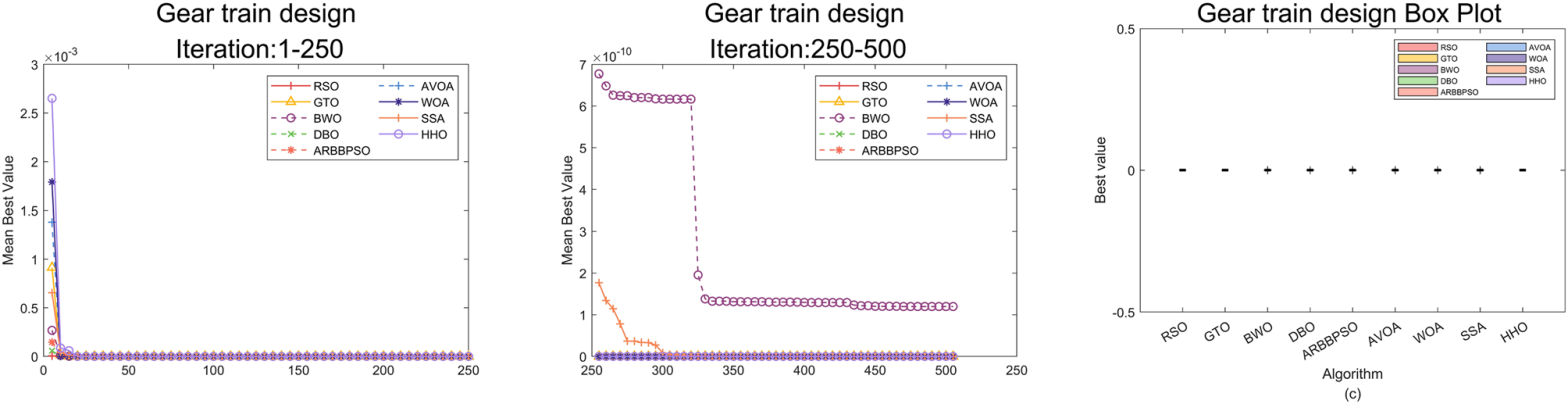
Convergence curves and error bars of RSO and the control group algorithms on GTD.

The experimental results of RSO and the control group on GTD are shown Tables 15 and 16. In Table 15, it is clearly that RSO, GTO and HHO obtain the same optimal value 0.0000E+00 with three different sets of decision variables. Furthermore, Table 16 presents the statistical analysis on the experimental results of RSO and the control group. RSO, GTO and HHO still achieve the optimal values among the four metrics. To visualize the optimization process of these algorithms on GTD, the convergence curves and error bars of RSO and the control group are shown in Fig. 11.

### Three-bar truss design

The 3-BTD is a classical problem from civil engineering. Its optimization objective is the weight minimization of the truss structure. The primary constraints are based on the stress limitations of each bar, ensuring the structure can safely withstand applied loads without exceeding material stress limits. The details of this problem are as follows:

Minimize:

12 $$\begin{aligned} f({\bar{x}}) = l (x_2+2\sqrt{2}x_1) \end{aligned}$$

Subject to:

13 $$\begin{aligned} & g_1({\bar{x}}) = \frac{x_2}{2 x_2 x_1 + \sqrt{2}x_1^2}P - \sigma \le 0, \\&g_2({\bar{x}}) = \frac{x_2+\sqrt{2}x_1}{2 x_2 x_1 + \sqrt{2}x_1^2}P - \sigma \le 0, \\&g_3({\bar{x}}) = \frac{1}{x_1 + \sqrt{2}x_2}P - \sigma \le 0, \\&l = 100, P = 2, \sigma =2. \end{aligned}$$

With bounds:

14 $$0 \le x_1, x_2 \le 1.$$

**Table 17:** Results analysis of 3-BTD, Part 1.

	$x_1$	$x_2$	cost
RSO	**7.8868E-01**	**4.0825E-01**	**2.638958E+02**
GTO	7.8869E-01	4.0821E-01	2.639066E+02
BWO	7.8820E-01	4.0961E-01	2.644397E+02
DBO	7.8871E-01	4.0815E-01	2.638963E+02
ARBBPSO	7.8867E-01	4.0827E-01	2.638960E+02
AVOA	7.8852E-01	4.0868E-01	2.639084E+02
WOA	7.8857E-01	4.0855E-01	2.651717E+02
SSA	7.8875E-01	4.0803E-01	2.638973E+02
HHO	7.8874E-01	4.0807E-01	2.639996E+02

Significant values are in bold.

**Table 18:** Results analysis of 3-BTD, Part 2.

	Worst	Mean	Best	Std
RSO	**2.638958E+02**	**2.638958E+02**	**2.638958E+02**	**8.339587E-08**
GTO	2.639475E+02	**2.638958E+02**	2.639066E+02	1.512048E-02
BWO	2.657238E+02	2.638970E+02	2.644397E+02	3.789302E-01
DBO	2.638995E+02	**2.638958E+02**	2.638963E+02	8.285605E-04
ARBBPSO	2.638965E+02	**2.638958E+02**	2.638960E+02	1.968597E-04
AVOA	2.639801E+02	2.638959E+02	2.639084E+02	1.818733E-02
WOA	2.828427E+02	2.638959E+02	2.651717E+02	3.264787E+00
SSA	2.639016E+02	**2.638958E+02**	2.638973E+02	1.574216E-03
HHO	2.644278E+02	**2.638958E+02**	2.639996E+02	1.435749E-01

Significant values are in bold.

**Figure 12:**
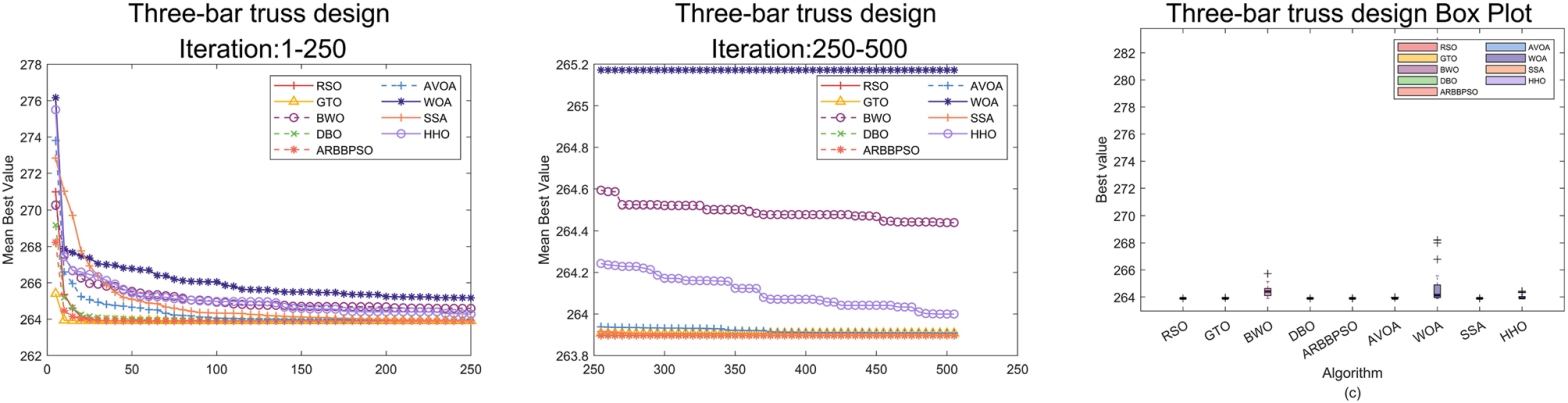
Convergence curves and error bars of RSO and the control group algorithms on 3-BTD.

The optimal results of the experiments on 3-BTD for RSO and the control group are shown in Table 17. The results indicate that RSO obtain the best solution with ${\bar{x}}$=(7.8868E-01, 4.0825E-01) and optimal value 2.638958E+02. Besides, Table 18 introduces the statistical analysis of the experimental results. Although the difference in the Mean of RSO, GTO, DBO, ARBBPSO, SSA and HHO across 36 independent experiments was small, RSO achieved the optimal values in the other three metrics. Particularly, the lowest Std indicates that RSO performs good robustness in solving 3-BTD problem.

To visualize the optimization process of these algorithms on 3-BTD, the convergence curves and error bars of HSO and the control group are shown in Fig. 12. RSO exhibits a significantly rapid downward trend in the early iterations. This suggests that RSO has the ability to rapidly approach the more optimal solutions at the early stage.

## Conclusions and future works

In this paper, we design a novel meta-heuristic optimization algorithm called Rhinopithecus Swarm Optimization (RSO). The proposed algorithm draws inspiration from the social behaviors of rhinopithecus. In RSO, we categorize the population into mature, adolescent and infancy rhinopithecus, each performing one of three search methods: vertical migration, concerted search, and mimicry, respectively. These search methods enhance global and local search capabilities, decreasing the possibility of falling into local optima in high-dimensional space.

To validate the performance of RSO, we conducted benchmark tests using 29 test functions from the CEC2017 and three classical engineering design problems. Furthermore, the Wilcoxon signed-rank and Friedman tests were used to analyze the experimental results. Eight well-known optimization algorithms were selected as the control group, including DBO, BWO, SSA, AVOA, WOA, ARBBPSO, GTO, and HHO. Both the experimental results and statistical analysis reveal that RSO achieves better accuracy than the control group. However, the proposed algorithm still has some limitations that need to be addressed. In particular, RSO performed worse than SSA and AVOA in Unimodal Functions with 30 dimension. Additionally, compared to ARBBPSO, RSO shows an insignificant advantage in Multimodal, Hybrid, and Composite Functions. Thus, enhancing its optimization capability in low-dimensional problems is a significant ongoing work. We also aspire to develop new search strategy for RSO to tackle multi-objective problems in the future.

## Data Availability

Data will be made available on reasonable request with the coorsponding author.

## Code availability

Code will be made available on reasonable request with the coorsponding author.
